# Enhancing nutrient recycling from excreta to meet crop nutrient needs in Sweden – a spatial analysis

**DOI:** 10.1038/s41598-019-46706-7

**Published:** 2019-07-16

**Authors:** Usman Akram, Nils-Hassan Quttineh, Uno Wennergren, Karin Tonderski, Geneviève S. Metson

**Affiliations:** 1 Theoretical Biology, Department of Physics, Chemistry and Biology, Linköping University, 581 83 Linköping, Sweden; 2 Department of Mathematics (MAI)/Optimization (OPT), Linköping University, 581 83 Linköping, Sweden; 3 Biology, Department of Physics, Chemistry and Biology, Linköping University, 581 83 Linköping, Sweden; 4 Center for Climate Science and Policy Research (CSPR), Linköping University, 581 83 Linköping, Sweden

**Keywords:** Ecological modelling, Environmental impact

## Abstract

Increased recycling of nutrient-rich organic waste to meet crop nutrient needs is an essential component of a more sustainable food system. However, agricultural specialization continues to pose a significant challenge to balancing crop nutrient needs and the nutrient supply from animal manure and human excreta locally. For Sweden, this study found that recycling all excreta (in 2007) could meet up to 75% of crop nitrogen and 81% of phosphorus needs, but that this would exceed crop potassium needs by 67%. Recycling excreta within municipalities could meet 63% of crop P nutrient needs, but large regional differences and imbalances need to be corrected to avoid over or under fertilizing. Over 50% of the total nitrogen and phosphorus in excreta is contained in just 40% of municipalities, and those have a surplus of excreta nutrients compared to crop needs. Reallocation of surpluses (nationally optimized for phosphorus) towards deficit municipalities, would cost 192 million USD (for 24 079 km of truck travel). This is 3.7 times more than the total NPK fertilizer value being transported. These results indicate that Sweden could reduce its dependence on synthetic fertilizers through investments in excreta recycling, but this would likely require valuing also other recycling benefits.

## Introduction

More sustainable nutrient management is essential to food security and to improve water quality globally^1,2^, and this dual importance has come to the forefront of European Union (EU) policies. First, losses of nitrogen (N) and phosphorus (P) to inland and coastal waters cause eutrophication, which may then lead to hypoxic conditions in aquatic ecosystems^3^. In the EU, legislation has been put in place to try to reduce nutrient losses from urban and rural areas to achieve “good ecological status” of all water bodies as stated in the Water Framework Directive^4^. In Sweden, and in other EU countries following the Urban Wastewater Treatment Directive, regulations to restrict wastewater nutrient discharges have gradually become more stringent as new environmental goals have been formulated. Though this has resulted in significant reductions in the anthropogenic load of N and P to e.g., the Baltic Sea^5^, nutrient enrichment continues to be a major issue^6^, and the sea still remains one of the largest nutrient-induced hypoxic zones in the world^7^. Further reduction in the nutrient load will require more focus on nutrient losses from agricultural areas. Part of those losses are related to the sub-optimal use of organic waste, particularly manure^8^. As animal husbandry farms have become larger, the manure is more concentrated in the landscape, which has often led to nutrient overapplication on fields close to where manure is produced and stored. This increases the risk for larger losses of both N and P to water bodies from those areas^8–10^, and estimating nutrient budgets at various scales is considered an essential component of efforts to reduce those losses^11^.

Second, even though N, P, potassium (K) and micronutrients are essential inputs to ensure high yields in agriculture, many farms are dependent on nutrient sources that are not renewable^12^. This includes synthetic N fertilizers produced using fossil fuels to fix atmospheric N into crop available N^13^ and P fertilizers produced from geopolitically concentrated phosphate rock deposits^14^. As such, mined P is subject to variability in price and physical availability on the global market^15^. In response, the EU has listed P as critical raw material^16^, which is a clear signal that the union welcomes management strategies that decrease food system vulnerability to fluctuations in the availability (physical or price) of synthetic P fertlizers. Historically, animal and human excreta recycling to supply crops with nutrients was a common and necessary agricultural practice, but agricultural specialization, urbanization and the availability of synthetic fertilizers have all contributed to less efficient recycling and a heavy dependency on synthetic fertilizers^9,15,17–20^. Finding ways to best utilize nutrient-rich organic waste^17,21^ will need to be an important part of sustainable nutrient mangement in the EU.

Other governance approaches are therefore needed to contribute to the EU objectives related to good nutrient management^22^. EU legislation regarding the use of human excreta (sewage sludge) regulates allowable concentrations of potential harmful substances, and does not explicitly focus on enhancing nutrient recycling to agricultural land^23^. Individual countries can however develop strategies for this. In Sweden, the Revaq certification of wastewater treatment plants was introduced to ensure active efforts to reach a low content of harmful substances in the sludge and work towards safe recycling of sludge P back to farmland^24^.

On the agricultural front, Sweden has decreased P surpluses over the past few decades, resulting in close-to-balanced national P budgets^25,26^, and a general decline in stream P concentrations^27^. Swedish legislation sets an upper limit of 22 kilogram (kg) P (over 5 yrs) and 170 kg N that can be applied from an organic source per hectare (ha) and year, and recommends that the P application rate be adapted to crop P uptake on soils rich in P-AL^28^. Still, the spatial separation of crop and livestock production, with high animal densities in some areas resulting in regional and local P surpluses, continues to be a significant cause of Sweden’s anthropogenic nutrient load to inland waters and the Baltic Sea^8,9^. Because transportation is a logistically complex and economically intensive endeavor, it is often viewed as a major barrier to effective recycling of organic waste and balancing agricultural landscape nutrient budgets^9,29,30^. To overcome this barrier and take full advantage of nutrient resources in organic waste and decrease the risk of nutrient losses from animal dense regions and cities, information on the spatial availability of nutrients from excreta and crop nutrient needs is required. More specifically, spatially explicit nutrient budgets with a higher resolution than the national level are needed to be able to explore country-wide transportation and logistical options^8,9,31^.

In this paper we quantify and map Sweden’s N, P, and K resources as animal and human excreta (together referred to simply as excreta in the rest of the text) and N, P, and K crop needs which are estimated based on fertilizer recommendations. We identify municipalities with current nutrient deficits and surpluses and use this information to explore recycling opportunities across Sweden. First, we aggregate municipal level data to determine the potential of excreta to meet crop nutrient needs nationally. This includes comparing nation-wide crop nutrient needs with current total nutrient supply in excreta and synthetic fertilizers, respectively, in order to better understand the potential to decrease synthetic fertilizer use by an enhanced recycling of excreta nutrients. Second, we examine the location and magnitude of municipal nutrient surpluses and deficits (excreta minus crop nutrient needs) to locate areas that would require excreta redistribution. Third, we use the locations of these surplus and deficit municipalities to estimate the distance and cost of transporting surplus excreta to deficit areas. We use a mathematical optimization model calibrated to minimize transport costs nationally based on P imbalances for this last step (referred to as the P optimization model). We chose to look at the distribution of P balances over N or K because P is emerging as a priority across the EU due to its dual importance to food security and environmental integrity^32^. We also run an optimization model to try to meet crop N, P and K needs simultaneously as an alternative to focusing on optimized P redistribution (referred to as the NPK optimization model). The methods used here are a modified version of the mass balance approach presented in Akram *et al*.^33^, (noting that what we refer to here as excreta is referred to as bio-supply in this previous manuscript). These modifications reflect the use of data with a better spatial resolution in the current paper. Our focus throughout the manuscript is on the potential of excreta to meet crop nutrient needs and as such we use the total amount of nutrients in excreta after storage losses. In the supplemental information (SI), we also present results based on the estimated crop available nutrients in excreta during the 1^st^ year of application.

## Results

### National nutrient supply and crop needs

**Figure 1:**
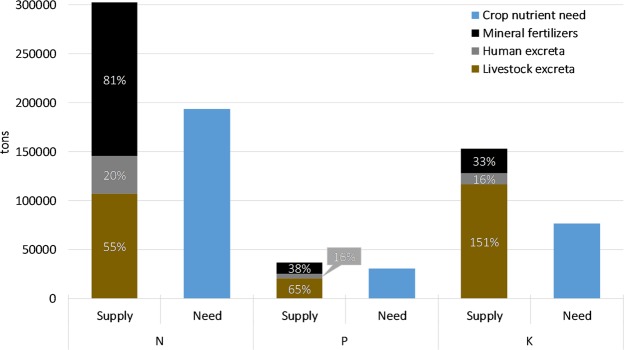
National 2007 Swedish nutrient supply and crop nutrient need. Nutrient supply sources (livestock and human excreta and synthetic fertilizers) are presented as both total amount of nutrients (y-axis) and as a percentage of total crop nutrient needs (white numbers in bars).

Nationally, synthetic fertilizers meet an important share of crop nutrient needs, but excreta could replace a substantial part of synthetic fertilizer use if crops are not overfertilized. The largest portion of crop nutrient needs come from ley hay, winter wheat, and spring barley, which together represent 70% of crop N needs (64% of P and K needs, Supplementary Table S1). Synthetic fertilizer use meets 81% of national N crop needs while accounting for a smaller proportion of P (38%) and K (33%) needs (Fig. 1). Excreta can meet up to 75% of N and 81% of P crop needs but represents a K surplus (67% over crop needs). The largest share of total nutrients in excreta comes from dairy cow manure (26% of N, 23% of P, and 30% of K), followed by humans, and other types of cows (heifers, bulls, and steers together, Supplementary Table S2). Adding synthetic fertilizers and excreta together results in a 110 000 tons N surplus (56% above crop needs), a 6 000 tons P surplus (20% above crop needs), and a 76,000 tons K surplus (double crop needs) across Sweden (Fig. 1). These surpluses represent 42 kg N/ha, 2 kg P/ha, and 30 kg K/ha of arable land in Sweden (i.e. 2578 thousand hectares).

### Municipal nutrient balances

**Figure 2:**
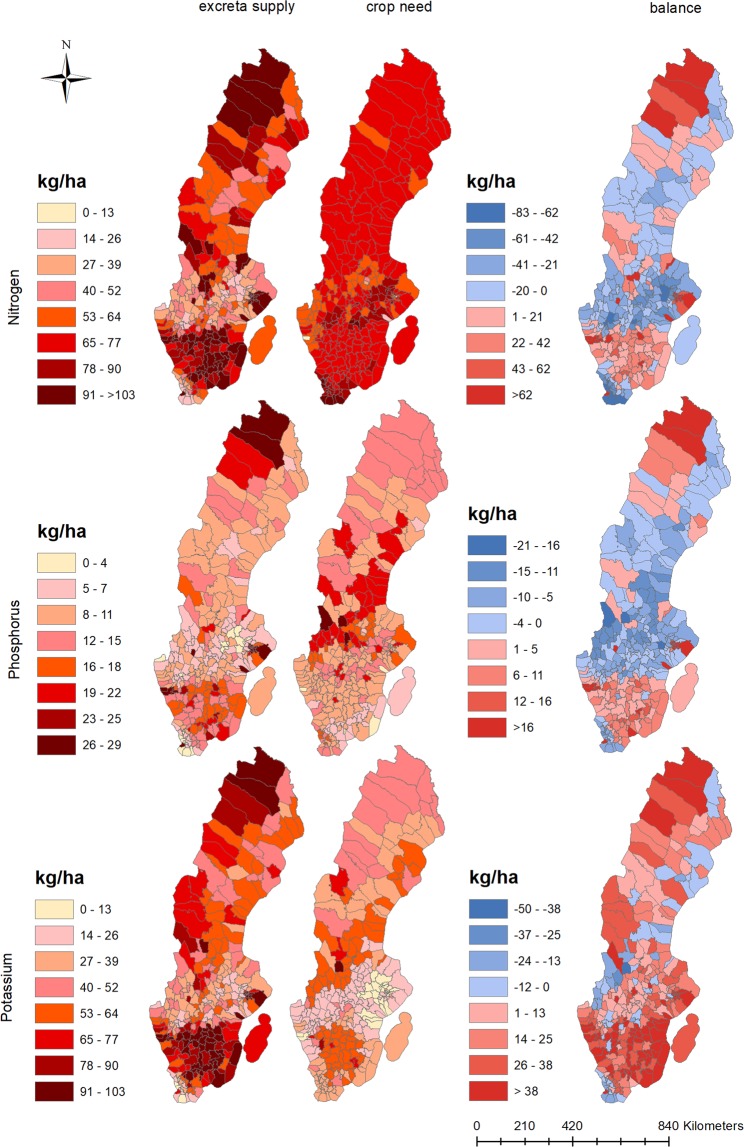
Spatial distribution of nutrients in excreta, crop nutrient needs, and nutrient balances of Swedish municipalities. The top panel represents N, the middle P, and the bottom K distributions. The right-side balance maps are created by subtracting crop nutrient need (middle) from excreta nutrient supply (left). Note that although the color scales are the same for all three nutrients, the values associated with each color are not (e.g., N values are much higher than for P).

**Figure 3:**
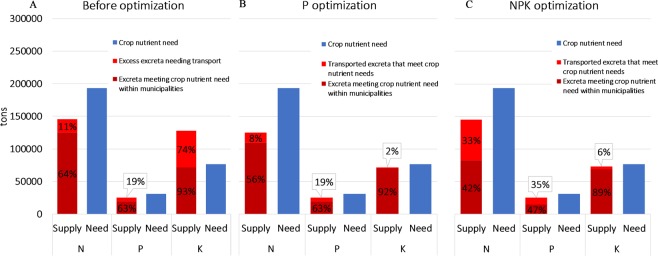
Total amount of nutrients in excreta that can be recycled within municipalities and transported between municipalities to meet crop needs. (**A**) The amount of crop nutrient needs that could theoretically be met by recycling before any optimization is run. (**B**) The amount of crop nutrient needs that could be met based on the redistribution made by the P optimization model. (**C**) The amount of crop nutrient needs that could be met based on the redistribution made by the NPK optimization model. Values are presented as the total amount of nutrients (y-axis) and as a percentage of total crop nutrient needs (black numbers in bars). Panels B and C only show the amount of excreta that meets crop nutrient needs, and not the surplus amounts within municipalities or resulting from excreta transport (all numbers are available in Supplementary Table S12).

Although there is less N and P in excreta nationally than crop N and P needs, there is a considerable amount of variation in municipal nutrient balances across the country (Fig. 2; Table 1). The majority of nutrients in excreta can be used locally to meet crop nutrient needs (within municipalities), but some excreta would require transportation between municipalities to avoid overapplication (Fig. 3A). Instead of expressing excreta supply and crop needs as total tons (as in Figs 1 and 3), we can express it as kilograms of nutrient per hectare of arable land to compare supply and need across municipalities. Nationally, average crop needs amount to 75 kg N/ha, 12 kg P/ha, and 30 kg K/ha, while the amount of nutrients in excreta is on average 56 kg N/ha, 10 kg P/ha, and 50 kg K/ha (the latter is 20 kg/ha above crop needs, Table 1). Municipal-scale crop needs and excreta supply deviate substantially from the national average (Table 1). For example, in the Solna municipality, there are no crop needs and a 17,000 times higher human excreta N supply than the national average, while the Vadstena municipality has high crop needs (103 kg N/ha) but only 27 kg of N/ha as excreta.

**Table 1:** Minimum, maximum and average crop nutrient needs and excreta supply in Swedish municipalities in 2007.

	N (kg/ha)			P (kg/ha)			K (kg/ha)		
	Min	Max	Avg.	Min	Max	Avg.	Min	Max	Avg.
Crop nutrient needs	0	103	75	0	29	12	0	103	30
Livestock excreta supply	0	135	42	0	24	8	0	159	45
Human excreta supply	2	262137	15	0	34825	2	1	82313	5
Total nutrients as excreta	15	262137	56	2	34825	10	7	82313	50

**Figure 4:**
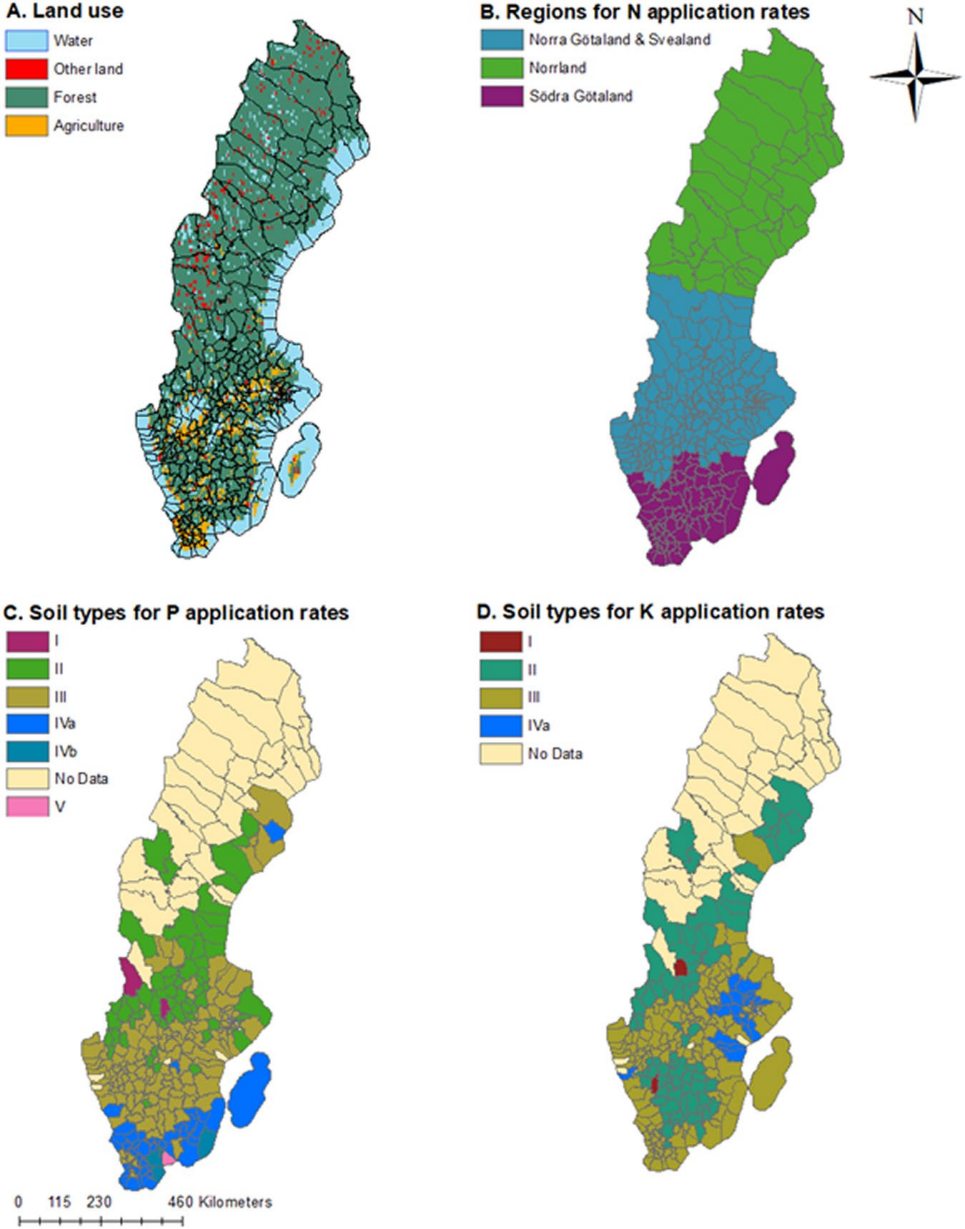
(**A**) Sweden’s land use^74^ and municipality delineations^75^. (**B**) division of Swedish municipalities into three production regions for N application rates^76^. (**C**) Division of Swedish municipalities into seven classes of P-AL^77^. (**D**) Division of Swedish municipalities into five classes of K-AL^77^.

Clustering of municipalities with surplus N and P as excreta is apparent in southern Sweden (except for the southernmost tip of the country which is a part of the Skåne Region) as well as in the very northern part of the country (Fig. 2). This pattern can be explained by higher crop nutrient needs (including intensive grain production) in the middle of the country (with the exception of a few municipalities in the south that also have high crop nutrient needs). Livestock production (and thus higher excreta nutrient supply) is located in the Southern part (with a few exceptions in the North). Although N, P, and K all generally exhibit this pattern, the distribution of nutrients in excreta and crop needs do exhibit slightly different spatial clustering for the three nutrients. For example, although Table (2) shows a similar level of spatial imbalances for N and P (e.g., the same percentage of surplus and deficit municipalities), mapping the distribution (Fig. 2) shows that the spatial distribution of P and K crop needs are influenced by soil classifications (Supplementary Table S4; Fig. 4C,D), while N needs reflect arable land distribution and crop choice more clearly. For example, the lower P crop needs in the majority of municipalities in Skåne (Fig. 2) is linked to higher soil concentrations of P-AL and thus lower fertilizer recommendations (Fig. 4C; Supplementary Tables S4, S15).

**Table 2:** Breakdown of municipalities with surplus and deficits of nutrients in relation to their share of arable land, crop nutrient needs, and nutrients in excreta at the national level.

Municipality balance		N		P		K	
		Surplus	Deficit	Surplus	Deficit	Surplus	Deficit
No. Of Municipalities		128	162	123	167	235	55
Arable land	(1000) ha	648	1931	871	1707	1995	584
	% of Total	25	75	34	66	77	23
Crop nutrient need	tons	44827	148841	7679	23309	55600	21322
	kg/ha	69	77	9	14	28	37
	% of total	23	77	25	75	72	28
Livestock excreta supply	tons	44506	62882	10861	9394	101940	14321
	kg/ha	69	33	12	6	51	25
	% of total	41	59	54	46	88	12
Human excreta Supply	tons	20974	17044	2730	2321	10343	1595
	kg/ha	32	9	3	1	5	3
	% of total	55	45	54	46	87	13
Total nutrients as excreta	tons	65480	79925	13591	11715	112283	15915
	kg/ha	101	41	16	7	56	27
	% of total	45	55	54	46	88	12
Net balance	tons	20653	−68916	5912	−11594	56683	−5407
	kg/ha	32	−36	7	−7	28	−9
	% of national crop need	11	−36	19	−37	74	−7

The net balance is the difference between crop needs and total nutrients in excreta, which is also expressed as a percentage of crop nutrient needs. These balances do not include the synthetic fertilizer supply which was only available at national scale.

In summary, much of Sweden’s crop nutrient needs can be met with excreta (Fig. 1), but the spatial concentration of supply is different from the spatial concentration of crop nutrient needs (Fig. 2). Therefore, we need to move excess excreta from surplus municipalities (Fig. 3A) to the deficit municipalities to ensure recycling actually meets crop nutrient needs. Recycling excreta *within* municipalities could meet up to 64% of N, 63% P, and 93% K crop needs in Sweden (Fig. 3A). The difference in the spatial availability of nutrients as excreta and crop nutrient needs result in 44% of municipalities having excreta surpluses of N, 42% having P surpluses, and 81% having K surpluses (Table 2). The total amount of excreta in municipalities with surpluses account for 45% of the excreta N and 54% of excreta P in the country, but only encompass 23% of N crop needs and 25% of P crop needs. Transporting excreta from surplus municipalities could meet an additional 11% of N, 19% of P, and 74% of K national crop needs (Fig. 3A and Table 2). In other words, transporting surplus nutrients within and between municipalities would fulfill a substantial amount of Sweden’s crop nutrient needs, up to 75% of N, 81% of P, and 100% of K crop needs.

### Transportation to redistribute excreta with the P optimization model

**Figure 5:**
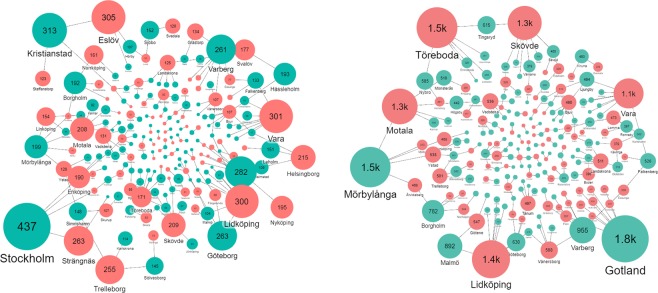
Transport network of P in excreta from surplus (sea green) to deficit (brick red) municipalities based on P optimization model outputs to minimize total national transport distance and eliminate surpluses of P. The left panel shows the amount (tons) of P from excreta exported or imported from or to a municipality where bubble size is proportional to the amount (also expressed as the number in the bubble). The right panel shows the distance (km) of P from excreta exported or imported from or to a municipality.

Transporting excess excreta from surplus municipalities towards deficit municipalities would require covering the costs associated with a minimum of 24 079 km of truck travel. The excreta associated with the P surplus in 123 municipalities (Table 2) represents 5.3 million tons (which contain 5 912 tons of P). There are 167 municipalities with P deficits, and they require an additional 11 594 tons of P to meet the crop need. Because there are more areas of deficit than surplus (and in larger quantities), only 85 of these deficit municipalities receive additional P from excreta in the nationally optimized model (Fig. 5; Supplementary Table S5). The average travel distance between a surplus and a deficit municipality to correct P imbalances would be 202 km (Table 3). On average a surplus municipality would export to two deficit municipalities (minimum one and maximum eight), while a deficit municipality would have two import connections (minimum one and maximum eight, Fig. 5; Table 3; Supplementary Table S5). Interestingly, the municipalities exporting or importing the largest amount of P are not necessarily those that require the largest transport distances (comparing Fig. 5 left and right panels). Stockholm and Kristianstad municipalities would be the top exporters based on the amount of P in excreta (they contain 13% of the P surplus nationally), but their total transport distances are relatively short, representing a total of 524 km (Fig. 5). Eslöv, Vara, and Lidköping would be top importers of P in excreta to meet crop needs (Fig. 5). The surplus of P from Gotland, Mörbylånga, and Varberg would require the most extended travel distances to reach P deficit municipalities (4 264 km or 18% of total cumulative transport kilometers), and Töreboda, Lidköping, Motala, and Skövde would cumulatively be receiving P from the longest transport routes to fulfill their P needs (Fig. 5; Supplementary Table S5).

**Table 3:** Summary values of transport amounts (tons of P in excreta) and distances (km) optimized to minimize costs (distance x fresh weight) to meet P crop needs.

	Export (tons)	Import (tons)	Export distance (km)	Import distance (km)	Export connections	Import connections
Min	1	1	12	31	1	1
Max	437	305	1789	1536	8	8
Average	50	70	202	283	2	2

Export/import connections represent the number of municipalities a surplus or deficit municipality would export to or import from.

We estimate that the cost of transporting surplus excreta to meet crop needs is 8.79 times the market value of P fertilizers it would replace, but only 3.68 times higher than the total NPK usable fertilizer value being transported. In other words, optimally transporting P in excreta would cost 192 million USD according to the model, while the same amount of P fertilizer would only cost 22 million USD^34^. However, the 15 783 tons of N and 1 233 tons of K that are transported along with the P also meet nutrient deficit needs and thus can replace further fertilizer use (Fig. 3B). Including the market value of N and K, the total market value of all three nutrients in the transported excreta is 52 million USD. It is important to note that there are regional differences in the relative costs associated with excreta transportation. For example, in the case of Stockholm, it only cost 29% of the fertilizer value of P to transport excreta (which is mostly human excreta) to deficit municipalities. Overall, the optimized redistribution of excreta could result in Sweden reducing purchases of synthetic fertilizer to 34% of N, 48% of P, and 17% of K of the current use of synthetic fertilizers in crop production (as tons) assuming all excreta nutrients eventually become crop available.

Although the transport of excreta from P surplus municipalities could correct many imbalances, most of the N and K that would be transported along with P was not done so optimally (comparing Fig. 3A,B values, Supplementary Table S12). After the redistribution of surplus excreta based on P crop needs, 193 municipalities had a balanced P budget (excreta supply equaled crop needs). These transports would also correct the balance of N and K for 9 and 8 municipalities respectively. However, in some cases redistributing surplus P would exacerbate or create N deficits. Fifteen of the P surplus municipalities had a N deficit before transport, while after transport 79 municipalities of the original P surplus municipalities were left with a N deficit. The majority of municipalities with a P deficit also had a N deficit (146 out of 165), but transport optimized for P resulted in 31 of these municipalities ending up with a N surplus. Only 3 of the P surplus municipalities were K deficient, but after transport 28 of these P surplus municipalities ended up with a K deficit. Finally, 51 of the P deficient municipalities also had a K deficiency, but after transport of surplus P excreta 28 of them ended up with a K surplus. In summary, 96% of K and 55% of N transported with surplus P had no fertilizer value (i.e., would be applied in a surplus quantity compared to crop needs, Supplementary Table S12).

### Transportation to redistribute excreta with the NPK optimization model

Transporting excreta to meet all three nutrient crop needs at once resulted in a larger amount of excreta being transported a longer distance than when optimized for only P, but would also meet a larger amount of crop nutrient needs (comparing Fig. 3B,C, Supplementary Table S12). The total transport distance would be more than double (53 463 km) and the amount of excreta transported also increased by a factor of 1.78 (9.5 tones) compared to the P optimization model outputs (Supplementary Table S12). More municipalities would achieve a balance between N and K excreta supply and crop needs, but less municipalities would achieve a P balance than when using the P optimization model (Supplementary Table S13).

Although more excreta is transported in the NPK model, the increased amount of transported nutrients that meet crop nutrient needs (in other words not resulting in overapplication) give a better ratio (1.27) of transport cost to market value for the transported excreta, compared to 3.68 from the P optimization model (Supplementary Table S12). Required transportation under the NPK optimization model would cost approximately 240 million USD. This is only 1.2 times the cost for the P optimization model, but the amount of transported nutrients that meet crop nutrient needs would be 4 times higher (Fig. 3C and Supplementary Table S12). Overall, using the results from the NPK optimization model would allow Sweden to use a lower amount of synthetic fertilizers, but the amounts are quite similar to the P optimization model. Sweden would still require 31% of the N, 48% of the P, and 13% of the K that was purchased in 2007 to meet crop nutrient needs.

## Discussion

At the national level, our findings of crop N and P needs, excreta and synthetic N and P content, and national balances are generally in line with existing literature (Supplementary Table S6), which indicate an excess of 39–45 kg N/ha and 0.5–4.1 kg P/ha and year^25–27,35–37^. The small value differences in total surpluses among studies, including ours, is likely linked to different system boundaries and nutrient balance calculation methods. For example, we used P fertilizer application recommendations adjusted for soil class based on soil P-AL content as opposed to estimating crop needs based on nutrients in harvested yields (as done in other studies^26,37^). Soil characteristics played an important role in our results as that influenced the estimated crop fertilization needs (the contribution from soil nutrients is often not considered an input in most national scale nutrient budget studies^25,37^). In our dataset, 25% of arable land had high P concentrations and, subsequently, these areas only represented 17% of the total crop fertilizer P need in Sweden (Supplementary Table S4). P surpluses were thus higher than the national average for these areas; as high as 20 kg/ha for the one municipality that had a surplus of P and was located in soil class V. Similarly, Stedje *et al*.^38^ found that the national P surplus in Norway was 5 kg/ha higher when crop P demand was adjusted for soil P status instead of just crop uptake. From a spatially explicit perspective, the surplus areas in the south of Sweden correspond to areas of high-density animal production and areas with high soil P and K concentrations (Figs 2 and 4C,D; Supplementary Fig. S1). This pattern is similar to nutrient-balance studies across Northern Europe where animal and crop production have been spatially segregated^38–41^. It should be mentioned that some of the nutrients in excreta will actually be applied to grazed agricultural land, and not just the arable grazed land considered in this study. We could not properly account for grazing on all agricultural land due to a lack of information on the grazing pressure, although we do not expect this such inclusion to drastically change the patterns we identified. For instance, a recent report indicated that, based on a sample inventory, 40% of the area classified as agricultural grazing land may have few, if any, grazing animals^42^. As better information on the use of this land becomes available, this should be incorporated in future nutrient balance studies. Finally, at the municipal scale, our range of P balances (−21 up to + 35 000 kg/ha; the plus maximum is in Solna where there is no crop need but a lot of human excreta) is wider than in similar Danish studies: −5 kg to 26 kg/ha for municipalities^43^, but similar to the range for parishes, −10 kg/ha to above 50 kg/ha^39^.

The municipal imbalances identified here, as well as the imbalances in the studies cited above, highlight the fact that 1) local recycling of excreta is not enough to ensure effective circular nutrient management and 2) that in most cases some synthetic fertilizer will still be required to meet crop needs. For Sweden, our results indicate that transporting excess nutrients in excreta can reduce synthetic fertilizer requirements further than just through local municipal recycling (Fig. 3). However, cost-effectiveness analyses of such transport depend on a number of assumptions. For instance, we used just one snap-shot price of mineral fertilizers and transportation costs and as these costs change in the future, so would the cost benefit ratio of recycling. If fertilizer prices were to increase by a factor of 3.7 then long-distance recycling in our model would be considered financially beneficial. Fertilizer prices have varied by this order of magnitude in the past. For instance, between 2005 and 2011, the highest global averaged annual price of P was 3.3 times the minimum price^44^, and the highest price payed by Swedish farmers for P (6.15 USD/kg in 2009) was 3.6 times the lowest price they paid in 2005^45^. However, because the snap-shot values we used were not historical lows, the real-world prices that would be required for cost-effectiveness can be considered quite high (e.g., 13.69 USD/kg for P). Another way to affect the cost-effectiveness ratio would be decreasing transportation costs. If fertilizer prices remain stable, then transportation costs would need to decrease by 73% to make recycling cost-effective in our model. Considering our parameter value for transport costs was conservative, such a drastic decrease is unlikely to be realistic without significant subsidies or a legal framework requiring nationally optimal recycling.

Another assumption example is how the P optimization model assumes that nutrients in excreta after storage and transport are fully crop available, which may be true over multiple years^46^. However, in the first year a lower percentage is likely available for crop uptake^47^. If we account for this, fewer municipalities have a P surplus in excreta (33 fewer), which in turn changes the optimal way surpluses would be redistributed (SI section 2; Tables S12 and S13). Despite this reduced potential to meet annual crop needs with excreta nutrients, the ratios of transport costs to fertilizer value of the excreta transported are quite similar: 3.7 for total nutrients vs 3.8  for crop available nutrients (Supplementary Table S12).

On the other hand, using the NPK optimization model instead of the P optimization model resulted in a better transport to fertilizer value ratio, and could reduce the need for synthetic fertilizers a little further, but caution must be taken in interpreting such a result. Because excreta is moved to minimize any overapplication of nutrients, the model produces a solution where excreta from one municipality is moved to another even if the ‘source’ municipality could have used the nutrients in excreta to meet local crop needs, i.e. more excreta is transported in total (compare Fig. 3B,C, Table S12). Although this maximizes the value of the nutrients in excreta it is impractical. In reality, farmers would likely avoid moving excreta and meet their own crop nutrient needs before transporting any excreta away. Therefore, the result from the P optimization model (ratio of 3.68) is probably more realistic.

These results contribute to the knowledge base required to move towards more effective recycling, but further work is needed. The spatially explicit nutrient balances and the optimization model we created indicate that a complete redistribution of surplus excreta (optimized for P) at the national level for Sweden does not seem favorable because total transport costs are higher than the value of transported fertilizers. With higher resolution spatial data, a more realistic picture of transportation costs could be determined because municipalities in Sweden are large and our current model does not account for within municipality transport needs or real road networks. Similarly, excreta processing technologies may affect the weight of excreta derived nutrients and their availability to crops and should be integrated in scenario development and model runs to more accurately produce cost-effectiveness analyses. This could also include technologies that allow for benefits beyond the nutrient value of excreta collection to be monetized (e.g., biogas, soil carbon storage or reduced risks for disease spread) as this could make excreta recycling more cost effective.

It may also make sense to explore locally or regionally optimized scenarios in addition to national ones. Transport might be more viable at the regional level. For example, our results highlight that exporting surplus nutrients from Stockholm only requires transporting to adjacent municipalities (Supplementary Table S5). In addition, the selection of any particular processing technology will depend on diverse local conditions including energy and fertilizer market prices at the time^48^, regulations, environmental priorities, and consumer and producer perceptions of sustainability, health and safety related to the reuse of organic waste (in particular human excreta^49^). Some of these considerations may be influenced by national policies and priorities, but they are also likely to vary across the country, which makes smaller scale models a relevant step forward.

As per the suggestions for moving forward with this work described above, policy makers have a number of options to intervene in making efficient recycling of excreta possible. These may vary for example from policies to promote mixed agricultural systems which reduce the distance between supply and demand areas for nutrients, valuing the processing of organic waste for multiple resources at once making inefficient local recycling unattractive, to changing the cost of synthetic fertilizers so that the price of recycling is more favorable. Regardless of the strategy selected however, there will be a need to move nutrients in waste back to where they are needed. As such advancing optimization models can be part of the solution.

## Methods

### Study area

Most of Sweden is forested, with only 6.5% of the country’s area in arable production^50^, and the country is divided into 290 municipalities^51^ (Fig. 4A). Because the growing season is 100 days longer in the South than in the North of Sweden, there is a gradient from more to less arable land as one moves North^50^ (Fig. 4A). Over the last two decades, Sweden’ agricultural sector has gone through large-scale structural changes; farms have become more specialized in crop production or animal rearing^50,52^. This spatial separation of crops and animals, in addition to urbanization separating people from the agricultural land, has also resulted in the separation of nutrient needs (from crops) from a recyclable nutrient supply (animal and human excreta). In this study, we consider this spatial distribution of supply and need of N, P, and K at the municipality scale. Municipalities represent an appropriate scale to examine the potential of nutrient recycling because municipal governments in Sweden are mandated to deal with local environmental issues as well as spatial planning, waste collection, and waste disposal^53^. Determining if municipalities should be importing or exporting excreta to meet crop nutrient needs could help them plan for how to move away from waste management to resource reuse and to make decisions on how to address issues associated with eutrophication.

### Calculating nutrient surpluses and deficits

To estimate the amount of nutrients in excreta in each municipality, we multiplied annual data on populations (livestock and human)^54,55^ with annual nutrient excretion rates per individual^56–58^, and subtracted gaseous losses during storage for N^59^. See Eq. (1), Table (4) and Supplementary Table S14) for more details on data sources. For national and regional estimates, we summed the municipal excreta values. Although not the main focus of our analyses, we did calculate crop available nutrients in excreta^47^ and conducted the same analyses as mentioned below with these lower bound numbers (Supplementary Table S14).

**Table 4:** Equations’ parameters, specifications, assumptions, and data sources used for nutrient balance calculations.

Eq.	Param.	Definition/Variables represent (Specifications, assumptions and data sources)
1	${Q}_{m}^{n}$	Total quantity of nutrient *n* in excreta in municipality *m*, where *n* represents nutrient (N, P or K), and $m\in M$ represents municipality. Here $m$ is the set of all municipalities.
	${E}_{lm}$	Total number of individuals, where $l\in L$ represents source (livestock type or human), and *m* represents municipality. Here *L* is the set of sources (i.e., livestock and human). The municipal total human population was obtained for the year 2007 for 290 municipalities from^54^ Municipal total livestock population for 2007 was obtained from^55^ for each of the following animal types: dairy cows, cows for calf production, heifers, bulls and steers, calves under 1 year, rams and ewes, lambs, breeding boars, breeding sows, fattening pigs 20 kg and over, piglets under 20 kg, poultry, laying chickens, broilers, turkeys and horses. Data for horses were not available for 2007, so we used these 2003 data^55^. Poultry and pigs achieve their final weight and are slaughtered in less than a year. Therefore, annual records collected annually do not reflect the entire year of production. However, once slaughtered these animals were replaced by new animals. This assumption allowed us to use annual excretion rates^56^.
	${e}_{l}^{n}$	Excretion per individual, where *n* represents nutrient (N, P or K), and *l* represents source (livestock type or human) We obtained the Swedish specific coefficients of N, P and K excretion for most livestock from^56^. The coefficients were given per animal per year. We obtained the nutrient excretion rates of fowls and breeding boars from^58^ We obtained the Swedish specific coefficients of N, P and K for human excreta from^57^. The coefficients were given per human per year.
	${v}_{l}^{n}$	Constant for a gaseous loss, where *n* represents nutrient (N, P or K), and *l* represents source (livestock type or human) We used the Swedish specific storage loss of N of manure from^59^. We assumed that manure from sheep, horses, broilers, and turkeys was stored as semisolid ($v$ = 0.2), while other kinds of manure were stored as a slurry ($v$ = 0.1). These storage losses are given for a covered storage with an opening for the pump. We assume the same storage loss for human excreta as we used for excreta stored as slurry.
2	${C}_{m}^{n}$	Total crop fertilizer needs in a municipality, where $n$ represents nutrient (N, P, and K), and *m* represents municipality
	${A}_{tm}$	Cropped area (hectares), where $t\in \Gamma$ represents crop/crop group, and $m$ represents municipality. Here $\Gamma$ is the set of crop groups. Municipal total arable land use for 2007 was obtained from^60^ for each of the following crops/crop groups: winter wheat, spring wheat, rye, winter barley, spring barley, oats, triticale, mixed grain, field peas for cooking, fodder peas, vetches and field beans, green peas, white beans, green fodder, utilized ley for hay and pasture, ley for seeds, table potatoes, potatoes for processing of starch, sugar beets, winter rape, spring rape, winter turnip rape, spring turnip rape, oil flax, horticulture plants, other crops, energy forest, fallow land, other untilled arable land, and unspecified arable land. Other crops include white mustard and oilseed crops^60^. We disaggregated hay and grazed pasture land uses on arable land by assuming the fraction of cut vs. grazed pasture at the county was representative of the municipalities it encompassed^65^. For Blekinge county, we used Skåne statistics. We assumed unspecified arable land was used to grow vegetables. Note that although we do include grazing land that is on arable land, we do not include grazing areas that are part of what is known as agricultural land but not arable land. This is because there are no good statistics on how much of this extensive, and usually low production land, is actively used and how many, and how intensively animals use it (or what type of animals).
	${R}_{tm}^{n}$	Recommended fertilizer rate of a nutrient per hectare, where $n$ represents nutrient (N, P, and K), and *t* represents crop/crop group, and *m* represents municipality We selected application rates that matched 2007 Swedish crop yields (Statistics Sweden, 2017b) for the following crops: Winter wheat, Spring wheat, Rye, Winter barley, Spring barley, Oats, Mixed grain, Peas, Field beans, Corn, Table potatoes, Potatoes for processing of starch, Sugar beets, Winter rape, Spring rape, Winter turnip rape, Spring turnip rape, Oil flax, Arable grassland We obtained agro-climate specific fertilizer recommendations of N, and soil class specific fertilizer recommendations of P_,_ and K from^56^. For the following crops, we had to use some other sources or a different strategy to select a fertilizer recommendation: Mixed grain = average of all grain crops, Green fodder = average of maize and ley hay, Oil flax = average of oilseed crops, Other crops = average of oilseed crops, Ley hey = We assumed a mixture of grasses and legumes (20% white clover) and 3 expected cuts, Ley seed^61^ where we use the average of all recommendations to different ley seeds, Horticulture plants^63^, Energy forests^62^, Vegetables^63^, where we use the average of all recommendations for different vegetables. In^56^ for Norrland N recommendation to winter wheat, rye oats and triticale were missing. For these crops, we used the same recommendations as they were given for Norra Götaland & Svealand. In *No data* soil classes of P-AL and K-AL, we used average fertilizer recommendations of a crop for all soil classes combined. Note that because we use fertilizer recommendations and not crop demand, we are implicitly accounting for N fixation by legumes. Because we are only looking at one year, we are not accounting for multi-crop or multi-year rotations (e.g., green fertilizers).
3	${B}_{m}^{n}$	Balance of nutrient *n* in municipality *m*, where *n* represent nutrient N, P, or K
4	SWE^*n*^	National balance of nutrient *n* for Sweden.
	SF^*n*^	Nutrient *n* sold in synthetic fertilizers at the national scale. We obtained synthetic fertilizer data at the national scale from^73^ which was given as total consumption of N, P and K in the country.

Although excretion rates for nutrient supply were only expressed as national averages, we were able to be more specific about crop nutrient need for different areas of Sweden. As in Akram *et al*.^33^, we opted to use fertilizer recommendations to calculate crop nutrient needs. Fertilizer recommendation rates should be designed to help achieve maximum yields, but also minimize the nutrient losses based on local biophysical and management conditions, including by taking advantage of potential legacies of past management (e.g., nutrient accumulation in soils^42^). We multiplied the annual arable area in a municipality^60^ with annual nutrient recommendations per hectare of a crop^56,61–63^ (see Eq. 2; Table 4; Supplementary Table S15), with recommended rates varying by area for all three nutrients (Fig. 4B–D). We assigned municipalities to production or soil class areas. Then we selected application rates that matched 2007 Swedish crop yields^64^ because fertilizer recommendations vary with expected yields^56^. More specifically, we aggregated municipalities into three production areas for N application rates, seven for P application, and five for K application rates. N fertilizer application rates were given for three regions: Norrland, Norra Götaland & Svealand together, and Södra Götaland^56^, (Fig. 4B; Supplementary Table S15). We categorized the municipalities into these production regions based on the agricultural research districts given in^41^ and merged the Östra and Västra districts into the Norra Götaland & Svealand region (Fig. 4B). P and K recommendations from^56^ were based on the concentrations of plant-available P and K (P-AL and K-AL), which were expressed as seven soil classes of P and five soil classes of K^56^. We categorized the municipalities into soil classes (Fig. 4C,D) using a gridded database where 13,000 samples were used to determine the concentration of P-AL and K-AL at a 10 km^2^ resolution^42^. We took the average P-AL and K-AL values of the points given within each municipality’s boundary to assign a soil class value of P-AL and K-AL to that municipality. Although most crop recommendations matched reporting categories for land use, recommendations for arable grassland such as cut hay and grazed pasture on arable land were given separately^56^, while reported farmland use is combined at the municipality level. To assign an area for each of these lands uses at the municipal level, we assumed that the fraction of cut hay vs. grazed pasture at the county scale^65^ was representative of the municipalities within that county. For national and regional estimates of crop nutrient need, we summed crop nutrient need of the municipalities.

We calculated municipal nutrient balances (see Eq. 3 and Table 4) where we subtract crop nutrient needs from total nutrients in excreta in each municipality. The sum of net surpluses (excreta supply > crop fertilizer recommendations) and deficit values of municipal nutrient balances then gave us the national nutrient balance. Finally, at the national scale, we added synthetic fertilizer use to the excreta nutrient supply (see Eq. 4 and Table 4) to be able to compare it with the crop nutrient need as well as the supply of excreta.

We use the mass balance approach to calculate potential nutrient surpluses and deficits at municipal scale (Eq. 3) and apply it more specifically to calculate municipal nutrient supply as manure and human excreta in Eq. (1) and municipal crop nutrient need in Eq. (2). These equations are the same as used in Akram *et al*.^33^ but with updated data sources. The national balance of nutrient *n* for Sweden is calculated in Eq. (4).

1 $${Q}_{m}^{n}=\sum _{l\in L}{E}_{lm}\,{e}_{l}^{n}(1-{v}_{l}^{n})$$

2 $${C}_{m}^{n}=\sum _{t\in \Gamma }{A}_{tm}{R}_{tm}^{n}$$

3 $${B}_{m}^{n}={Q}_{m}^{n}-{C}_{m}^{n}$$

4 $${{\rm{SWE}}}^{n}=\sum _{m\in M}{B}_{m}^{n}+{{\rm{SF}}}^{n}$$

As equations in display mode are not allowed per style in the Table, the original equations (1, 2, 3, 4, 5, 6 and 7) given in Table 4 and 5 has been made run-in with the Body text.

### Calculating transport distances for surplus excreta

To estimate transport distances, we first chose to look at the distribution of P balances over N or K because P is emerging as a priority across the EU due to its dual importance to food security and environmental integrity^32^. This model is refered to here as the P optimization model (P-Opt). After presenting this model we discuss an alternative NPK optimization model (NPK-Opt) which accounts for all three nutrients at once. Finally we compare the assumptions and contraints in both models. Except when specified, both models use the same input data, transformation steps, and analysis metrics.

We used ArcMap 10.3.1 to merge the municipal boundaries and municipal nutrient supply in excreta, crop needs, and nutrient balances in order to represent these values spatially. We then calculated the distance (dist_*ij*_) between centroids (centers) of all municipalities to determine the paths available to link surplus municipalities *i* to deficit municipalities *j* for a given nutrient. To determine how much excreta should move from one municipality to another, we modeled and solved two versions of the Transportation Problem, a well-known optimization problem formulated and described by Hitchcock in 1941^66^. The objective function is to minimize the total costs associated with transports of excreta. We implemented the model in AMPL^67^ and used the commercial solver cplex^68^. The modeled solution provides the total transport cost in United States Dollars (USD). Transport costs were based on the actual weight of manure from livestock and weight of excreta (dry mass) from humans associated with the amount of nutrients that needed to move and the transport distances. We use a weighted average of nutrients to tons of excreta conversion based on the average mix of the excreta for a specific municipality (Eq. 5 and 6). In order to estimate the amount of N and K in excreta transported along with P (in P optimization model), we used Eq. (7).

**Table 5:** Equations’ parameters, specifications, assumptions, and data sources used for the optimization models where we analyze the transport of surplus excreta towards municipalities with a nutrient deficit to meet crop needs.

Eq.	Param.	Definition/Variables represent (Specifications, assumptions and data sources)
5	${W}_{i}$	Total weight of excreta in municipality $i\in S$
	*g*_*l*_	Weight of excreta per individual and year from source *l*, human sludge or manure. We obtained the coefficient (m3) of slurry and solid manure production of livestock from[58] and human excreta production (as dry mass) per year from^57^. A m3 of solid manure weighs 0.5 tons, and a m3 of slurry weighs 1 ton[58]
	*E*_*li*_	Number of individuals of source *l* in municipality *i*
6	${k}_{i}^{n}$	Concentration, the amount of nutrient *n* in each ton of excreta at each surplus municipality *i* which is used in constraint 8.2
	${Q}_{i}^{n}$	Total quantity of nutrient*n* in excreta in municipality *i*, where *n* represents nutrient (N, P or K), (Eq. 1)
7	${T}^{n}$	Total amount of nutrient *n* transported along with a surplus of P
	${B}_{i}^{n}$	Balance of nutrient *n* in municipalities *i*, where *n* represent nutrient N, P, or K (Eq. 3)
	${Q}_{i}^{n}$	Total quantity of nutrient *n* in excreta in municipality *i* (Eq. 1)

We could then compare the cost of transportation with the fertilizers value of what was transported, as well as compare these values to existing expenditures on synthetic fertilizers^34^. Note that we only used the value of ″useful″ nutrients which were transported; in other words we did not put a dollar value on the amounts of N and K that when transporting excess P optimally through the P optimization model, would result in over-application of N and K in the receiving municipality. We applied the same method with excess K moving among municipalities when using the NPK optimization model. Note that for any optimization problem there can be alternative optimal solutions which all produce the same optimal objective value (where the objective value is cost). In other words, there could be different transportation patterns and amount of excreta transported that cost the same thing.

Eqs (5) and (7) are valid for all supply municipalities $i\in S$, where $S=\{m\in M\,:\,{B}_{m}^{{\rm{P}}} > 0\}$.

5 $${W}_{i}=\sum _{l\in L}{E}_{li}{g}_{l}$$

6 $${k}_{i}^{n}=\frac{{Q}_{i}^{n}}{{W}_{i}}$$

7 $${T}^{n}=\sum _{i\in S}\frac{{B}_{i}^{{\rm{P}}}}{{Q}_{i}^{{\rm{P}}}}{Q}_{i}^{n}$$

### P optimization model

It is not possible to meet all crop P needs at the national scale, and therefore we enforce that all supply must be distributed while the need at each municipality should be respected (used as an upper bound). As such for each municipality, the supply and need of P are pre balanced within the municipality before any optimization takes place. All municipalities are then classified as supply or demand nodes, based on parameter ${B}_{m}^{n}$ from Eq. (3).

Following this balancing, set $S=\{m\in M:{B}_{m}^{{\rm{P}}}* * * * * 0\}$ represents all supply municipalities, and parameter *s*_*i*_ = *W*_*i*_ is the corresponding amount of excreta (which contains the surplus of P) to be transported away from municipality $i\in S$. Similarly, set $D=\{m\in M:{B}_{m}^{{\rm{P}}}* * * * 0\}$ represents all demand municipalities, and parameter ${d}_{j}^{{\rm{P}}}=-\,{B}_{j}^{{\rm{P}}}$ is the demand of nutrient P for municipality $j\in D$. Variable *x*_*ij*_ is the amount (in tons) of excreta sent from a supply municipality $i\in S\subseteq M$ to a demand municipality $j\in D\subseteq M$.

8 $$[{\rm{P}}-{\rm{Opt}}]\,\min \,z=uf\sum _{i\in S}\sum _{j\in D}{{\rm{dist}}}_{ij}{x}_{ij}$$

subject to

8.1 $${\sum }_{j\in D}{x}_{ij}={s}_{i},\,i\in S$$

8.2 $${\sum }_{i\in S}{k}_{i}^{{\rm{P}}}{x}_{ij}\le {d}_{j}^{{\rm{P}}},\,j\in D$$

8.3 $$\,{x}_{ij}\ge 0,\,\,i\in S,j\in D$$

Objective 8 is to minimize the total costs for distributing the excreta. Parameter *u* is a unit cost for transportation of excreta, here 0.25 USD (2 SEK per ton per km, which is based on liquid manure transport costs in Sweden specifically)^69^, and parameter *f* is a distance factor to approximate the actual road distances (we used 1.33^70^). Constraint 8.1 makes sure that the total amount of P sent from each supply node is equal to the stated supply. The transportation cost and approximation of road distances are comparable to other studies and remain on the conservative side as our parameter values did not include road choice preferences, manure spreading on fields, loading and unloading of trucks, or solid manure handling all of which increase costs per ton and distances traveled^71,72^.

Parameter ${k}_{i}^{{\rm{P}}}\,$from Eq. (6) represents the concentration, that is, the amount of nutrient P in each ton of excreta at each surplus municipality i, which is used in constraint 8.2. Constraint 8.2 makes sure that P forwarded to each demand node does not violate the requested demand. Constraint 8.3 makes sure that all flows are non-negative.

### NPK optimization model

In the NPK optimization model, it is possible for a municipality to simultaneously be a supply and a demand node. In contrast to the P optimization model, nutrient supply and demand is not balanced out before the optimization takes place, instead the input to the model is the total amount of nutrients in excreta and the total amount of nutrients needed by crops in each municipality.

Like in the P optimization model, the supply parameter *s*_*i*_ is the total excreta (in tons) for each municipality *i*, while demand parameters ${d}_{j}^{{\rm{N}}}$, ${d}_{j}^{{\rm{P}}}$, and ${d}_{j}^{{\rm{K}}}$ are the deficits of nutrients N, P, and K (in tons), respectively, for each municipality *j*.

9 $$[{\rm{NPK}}-{\rm{Opt}}]\,\,\min \,z=uf\sum _{i\in S}\sum _{j\in D}{{\rm{dist}}}_{ij}{x}_{ij}$$

subject to

9.1 $${\sum }_{j\in D}{x}_{ij}\,=\,{s}_{i},\,i\in S$$

9.2 $${\sum }_{i\in S}{k}_{i}^{{\rm{N}}}{x}_{ij}\le {d}_{j}^{{\rm{N}}},\,j\in D$$

9.3 $${\sum }_{i\in S}{k}_{i}^{{\rm{P}}}{x}_{ij}\le {d}_{j}^{{\rm{P}}},\,j\in D$$

9.4 $${\sum }_{i\in s}{k}_{j}^{{\rm{K}}}{x}_{ij}\le {d}_{j}^{{\rm{K}}}\,j\in D$$

9.5 $${x}_{ij}\ge 0,\,i\in S,j\in D$$

The total amount of nutrient K in excreta is far more than the need for K. Hence, in order to find feasible solutions, constraint 9.4 must be relaxed. We have multiplied the right-hand side of the equation by a factor of three for K here (constraint 9.4).

The exact relationship between the P optimization model and the NPK optimization model is not trivial because of the following reasons:

- Because of the pre balancing of nutrient P in [P-Opt], it is a restriction of [NPK-Opt] with respect to this assumption.
- But at the same time, constraints 9.2 and 9.4 are not part of [P-Opt], hence it is a relaxation of [NPK-Opt].
- If constraints 9.2 and 9.4 are removed, one might think that solving this relaxed version of [NPK-Opt] is equivalent to solving [P-Opt] since we only take nutrient P into consideration. But the pre-balancing taking place before solving [P-Opt] is equivalent to removing all variables *x*_*ij*_ in [NPK-Opt] where indices *i* and *j* correspond to the same municipality. This is the same as fixing all those variables to 0, hence a restriction.

## Supplementary information

Enhancing nutrient recycling from excreta to meet crop nutrient needs in Sweden – a spatial analysis
