# Author Correction: Enhancing nutrient recycling from excreta to meet crop nutrient needs in Sweden – a spatial analysis

**DOI:** 10.1038/s41598-019-55234-3

**Published:** 2020-01-10

**Authors:** Usman Akram, Nils-Hassan Quttineh, Uno Wennergren, Karin Tonderski, Geneviève S. Metson

**Affiliations:** 1 Theoretical Biology, Department of Physics, Chemistry and Biology, Linköping University, 581 83 Linköping, Sweden; 2 Department of Mathematics (MAI)/Optimization (OPT), Linköping University, 581 83 Linköping, Sweden; 3 Biology, Department of Physics, Chemistry and Biology, Linköping University, 581 83 Linköping, Sweden; 4 Center for Climate Science and Policy Research (CSPR), Linköping University, 581 83 Linköping, Sweden

Correction to: *Scientific Reports* 10.1038/s41598-019-46706-7, published online 16 July 2019

The original version of this Article contained an error in the Abstract.

“For Sweden, this study found that recycling all excreta (in 2007) could meet up to 75% of crop nitrogen and 81% of phosphorus needs, but that this would exceed crop potassium needs by 51%.”

now reads:

“For Sweden, this study found that recycling all excreta (in 2007) could meet up to 75% of crop nitrogen and 81% of phosphorus needs, but that this would exceed crop potassium needs by 67%.”

These errors have now been corrected in the PDF and HTML versions of the Article.

