# Supplementary material for: Enhancing nutrient recycling from excreta to meet crop nutrient needs in Sweden – a spatial analysis
Source: Sci Rep. 2019 Jul 16;9:10264. doi: 10.1038/s41598-019-46706-7 (PMC6635506; doi:10.1038/s41598-019-46706-7)
Supplement: Supplementary file 1 — Enhancing nutrient recycling from excreta to meet crop nutrient needs in Sweden – a spatial analysis [file 41598_2019_46706_MOESM1_ESM.docx]

# Enhancing nutrient recycling from excreta to meet crop nutrient needs in Sweden – a spatial analysis

Authors: Usman Akram^a^, Nils-Hassan Quttineh^b^, Uno Wennergren^a^, Karin Tonderski^c^, Geneviève S. Metson^ad*^

^a^ Theoretical Bioloy, Department of Physics, Chemistry and Biology, Linköping University, 581 83 Linköping, Sweden

^b^ Department of Mathematics (MAI) / Optimization (OPT), Linköping University, 581 83 Linköping, Sweden

^c^ Biology, Department of Physics, Chemistry and Biology, Linköping University, 581 83 Linköping, Sweden

^d^ Center for Climate Science and Policy Research (CSPR), Linköping University, 581 83 Linköping, Sweden

*corresponding author: [genevieve.metson@liu.se](mailto:genevieve.metson@liu.se)

**Supplementary Information**

**Overview:**

Section 1: We provide supplementary information for the P optimization model results presented in the main manuscript which use the total nutrient content of excreta.

Section 2: We provide comparative results to the P optimization model where we considered only crop available nutrients in excreta instead of the total.

Section 3: We provide comparative results for the two sections above using the NPK optimization model which considers not only balancing P supply and need in municipalities, but also N and K.

Section 4: We provide the supplementary information for methods section.

**Section 1: Supplementary information for the results of the main analysis**

**Supplementary Table S1.** Cultivated area and nutrient needs by crops in 2007 in Sweden expressed as hectares and tons of nutrients and percentage (%) of the total area and of the total nutrient need.

| Crop | Area (000 ha) | % Area | N (tons) | % N crop need | P (tons) | % P crop need | K (tons) | % K crop need |
| --- | --- | --- | --- | --- | --- | --- | --- | --- |
| Ley Hay | 833 | 32 | 66615 | 34 | 10796 | 35 | 34463 | 45 |
| Winter Wheat | 323 | 13 | 45032 | 23 | 5544 | 18 | 8440 | 11 |
| Spring Barley | 318 | 12 | 23998 | 12 | 3614 | 12 | 6477 | 8 |
| Oats | 208 | 8 | 14120 | 7 | 2731 | 9 | 4196 | 5 |
| Winter Rape | 50 | 2 | 5729 | 3 | 883 | 3 | 1465 | 2 |
| Green Fodder | 46 | 2 | 5324 | 3 | 443 | 1 | 3107 | 4 |
| Triticale | 53 | 2 | 5216 | 3 | 760 | 2 | 1323 | 2 |
| Sugar Beets | 41 | 2 | 4871 | 3 | 946 | 3 | 2206 | 3 |
| Ley Pasture | 190 | 7 | 4269 | 2 | 1218 | 4 | 4753 | 6 |
| Spring Wheat | 38 | 1 | 3976 | 2 | 447 | 1 | 622 | 1 |
| Spring Rape | 32 | 1 | 2915 | 2 | 509 | 2 | 413 | 1 |
| Rye | 24 | 1 | 2188 | 1 | 384 | 1 | 707 | 1 |
| Energy Forest | 14 | 1 | 2183 | 1 | 259 | 1 | 576 | 1 |
| Table Potatoes | 20 | 1 | 1625 | 1 | 993 | 3 | 3344 | 4 |
| Mixed Grain | 15 | 1 | 1365 | 1 | 154 | 0 | 268 | 0 |
| Unspecified Arable Land | 8 | 0 | 877 | 0 | 238 | 1 | 1151 | 1 |
| Ley Seeds | 13 | 1 | 662 | 0 | 182 | 1 | 620 | 1 |
| Potatoes for Starch Processing | 8 | 0 | 632 | 0 | 253 | 1 | 1287 | 2 |
| Winter Barley | 8 | 0 | 603 | 0 | 79 | 0 | 203 | 0 |
| Horticulture Plants | 15 | 1 | 518 | 0 | 31 | 0 | 268 | 0 |
| Other Crops | 6 | 0 | 490 | 0 | 92 | 0 | 91 | 0 |
| Oil Flax | 4 | 0 | 220 | 0 | 45 | 0 | 36 | 0 |
| Spring Turnip Rape | 2 | 0 | 192 | 0 | 35 | 0 | 30 | 0 |
| Winter Turnip Rape | 1 | 0 | 50 | 0 | 8 | 0 | 4 | 0 |
| Peas, Vetches, And Beans | 19 | 1 | 0 | 0 | 248 | 1 | 545 | 1 |
| Green Peas | 9 | 0 | 0 | 0 | 94 | 0 | 310 | 0 |
| White Beans | 1 | 0 | 0 | 0 | 2 | 0 | 16 | 0 |
| Fallow Land | 281 | 11 | 0 | 0 | 0 | 0 | 0 | 0 |
| Other Untilled Arable Land | 2 | 0 | 0 | 0 | 0 | 0 | 0 | 0 |

**Supplementary Table S2.** Total nutrients in excreta by Swedish livestock and human populations in 2007 expressed as tons of nutrients and percentage (%) of total supply for each source.

| Animal | Number (000) | % Population | N (Tons) | % N Supply | P (Tons) | % P Supply | K (Tons) | % K Supply |
| --- | --- | --- | --- | --- | --- | --- | --- | --- |
| Dairy Cows | 370 | 1 | 38735 | 27 | 5886 | 23 | 38257 | 30 |
| Human | 9183 | 35 | 38017 | 26 | 5051 | 20 | 11938 | 9 |
| Heifers, Bulls and Steers | 516 | 2 | 21800 | 15 | 4123 | 16 | 27829 | 22 |
| Cows for Calf Production | 186 | 1 | 10516 | 7 | 2226 | 9 | 13910 | 11 |
| Fattening Pigs, 20 Kg and Over | 1015 | 4 | 9663 | 7 | 2245 | 9 | 4197 | 3 |
| Calves, Under 1 Year | 489 | 2 | 9231 | 6 | 1465 | 6 | 13675 | 11 |
| Rams and Ewes | 242 | 1 | 5685 | 4 | 1015 | 4 | 9644 | 8 |
| Breeding Sows | 179 | 1 | 5452 | 4 | 1683 | 7 | 2188 | 2 |
| Horses | 95 | 0 | 3665 | 3 | 859 | 3 | 5536 | 4 |
| Poultry | 5328 | 20 | 936 | 1 | 319 | 1 | 319 | 0 |
| Broilers | 6653 | 25 | 932 | 1 | 250 | 1 | 457 | 0 |
| Laying Chickens | 1753 | 7 | 575 | 0 | 160 | 1 | 209 | 0 |
| Breeding Boars | 2 | 0 | 45 | 0 | 10 | 0 | 18 | 0 |
| Turkeys | 101 | 0 | 35 | 0 | 15 | 0 | 20 | 0 |

**Supplementary Table S3**. Total amount of nutrients in excreta (livestock manure and human excreta), crop nutrient needs, and nutrient balances for Swedish counties in 2007 expressed as kg/ha.

|  | N (kg per ha) | | | | | P (kg per ha) | | | | | K (kg per ha) | | | | |
| --- | --- | --- | --- | --- | --- | --- | --- | --- | --- | --- | --- | --- | --- | --- | --- |
| County | Crop need | Livestock excreta supply | Human Excreta supply | Total excreta-supply | Bala-nce | Crop need | Livestock excreta supply | Human Excreta supply | Total excreta-supply | Bala-nce | Crop need | Livestock excreta supply | Human Excreta supply | Total excreta-supply | Bala-nce |
| Stockholms | 72 | 21 | 102 | 123 | 51 | 14 | 4 | 14 | 18 | 4 | 13 | 26 | 32 | 58 | 45 |
| Uppsala | 79 | 20 | 8 | 28 | -51 | 11 | 4 | 1 | 5 | -6 | 8 | 22 | 3 | 25 | 17 |
| Södermanlads | 77 | 30 | 9 | 39 | -38 | 13 | 6 | 1 | 7 | -6 | 14 | 32 | 3 | 35 | 21 |
| Östergötlands | 84 | 40 | 9 | 49 | -35 | 12 | 8 | 1 | 9 | -3 | 21 | 43 | 3 | 46 | 25 |
| Jönköpings | 71 | 80 | 16 | 96 | 25 | 11 | 14 | 2 | 16 | 5 | 58 | 95 | 5 | 100 | 42 |
| Kronobergs | 68 | 78 | 15 | 93 | 25 | 10 | 14 | 2 | 16 | 6 | 59 | 91 | 5 | 95 | 36 |
| Kalmar | 74 | 75 | 8 | 83 | 9 | 8 | 14 | 1 | 15 | 7 | 31 | 83 | 2 | 86 | 55 |
| Gotlands | 74 | 56 | 3 | 59 | -15 | 7 | 10 | 0 | 11 | 4 | 27 | 64 | 1 | 65 | 38 |
| Blekinge | 71 | 76 | 20 | 96 | 25 | 8 | 16 | 3 | 18 | 10 | 43 | 76 | 6 | 82 | 39 |
| Skåne | 84 | 38 | 11 | 49 | -35 | 11 | 8 | 1 | 9 | -2 | 34 | 37 | 3 | 41 | 7 |
| Hallands | 68 | 70 | 11 | 81 | 13 | 9 | 14 | 1 | 15 | 6 | 28 | 67 | 3 | 70 | 42 |
| Västra Götalands | 73 | 40 | 14 | 54 | -19 | 13 | 8 | 2 | 9 | -4 | 29 | 43 | 4 | 47 | 18 |
| Värmlands | 66 | 30 | 11 | 41 | -25 | 15 | 6 | 1 | 7 | -8 | 37 | 35 | 3 | 38 | 1 |
| Örebro | 73 | 26 | 11 | 37 | -36 | 13 | 5 | 1 | 7 | -6 | 25 | 29 | 3 | 32 | 7 |
| Västmanlands | 72 | 19 | 11 | 29 | -43 | 14 | 4 | 1 | 5 | -9 | 12 | 19 | 3 | 22 | 10 |
| Dalarnas | 64 | 36 | 19 | 55 | -9 | 16 | 6 | 3 | 9 | -7 | 37 | 43 | 6 | 49 | 12 |
| Gävleborgs | 68 | 38 | 17 | 55 | -13 | 19 | 7 | 2 | 9 | -10 | 42 | 46 | 5 | 51 | 9 |
| Västernorrlad | 68 | 43 | 21 | 64 | -4 | 17 | 8 | 3 | 10 | -7 | 43 | 50 | 7 | 57 | 14 |
| Jämtlands | 71 | 48 | 13 | 60 | -11 | 16 | 8 | 2 | 10 | -6 | 51 | 57 | 4 | 61 | 10 |
| Västerbottes | 66 | 43 | 16 | 59 | -7 | 10 | 7 | 2 | 10 | 0 | 54 | 47 | 5 | 52 | -2 |
| Norrbottens | 71 | 41 | 31 | 72 | 1 | 12 | 7 | 4 | 11 | -1 | 39 | 47 | 10 | 56 | 17 |

**Supplementary Table S4**. Arable land, crop nutrient need, excreta supply (livestock and human excreta) and nutrient balance according to production regions and P-AL and K-AL soil classes in 2007 expressed as a percentage (%) of the national values and kg/ha.

| N | **N Region** | % of total arable land | % of total crop nutrient need | % of total supply in livestock excreta | % of total supply in human excreta | % of total excreta-supply | Crop nutrient need kg/ha | Supply in livestock excreta kg/ha | Supply in human excreta kg/ha | Total excreta-supply kg/ha | Nutrient balance kg/ha |
| --- | --- | --- | --- | --- | --- | --- | --- | --- | --- | --- | --- |
|  | Norra Götaland & Svealand | 56,5 | 55,9 | 43,9 | 63,9 | 49,2 | 74 | 32 | 17 | 49 | -25 |
|  | Norrland | 7,3 | 6,6 | 7,6 | 9,5 | 8,1 | 68 | 44 | 19 | 63 | -5 |
|  | Södra Götaland | 36,2 | 37,5 | 48,5 | 26,7 | 42,8 | 78 | 56 | 11 | 67 | -11 |
|  | **P-Al Class** |  |  |  |  |  |  |  |  |  |  |
| P | I | 0,2 | 0,4 | 0,1 | 0,2 | 0,1 | 28 | 5 | 3 | 8 | -20 |
|  | Ii | 15,8 | 25 | 13,2 | 14,6 | 13,5 | 19 | 7 | 2 | 8 | -11 |
|  | Iii | 55,6 | 54 | 49,7 | 60 | 51,8 | 12 | 7 | 2 | 9 | -3 |
|  | Iva | 21 | 15,1 | 27,4 | 14,5 | 24,8 | 9 | 10 | 1 | 12 | 3 |
|  | Ivb | 3,4 | 1,5 | 5,4 | 1,3 | 4,6 | 5 | 13 | 1 | 13 | 8 |
|  | V | 0,3 | 0,1 | 0,8 | 0,2 | 0,7 | 5 | 24 | 1 | 25 | 20 |
|  | No Data | 3,8 | 3,8 | 3,3 | 9,2 | 4,5 | 12 | 7 | 5 | 12 | 0 |
| K | **K-Al Class** |  |  |  |  |  |  |  |  |  |  |
|  | I | 0,3 | 0,9 | 0,5 | 0,2 | 0,4 | 93 | 71 | 3 | 74 | -19 |
|  | Ii | 18,4 | 35,4 | 28,3 | 18,7 | 27,4 | 58 | 70 | 5 | 74 | 16 |
|  | Iii | 65,1 | 57,6 | 60,9 | 46,1 | 59,5 | 26 | 42 | 3 | 45 | 19 |
|  | Iva | 12,5 | 1,4 | 6,7 | 25,8 | 8,5 | 3 | 24 | 10 | 34 | 31 |
|  | No Data | 3,8 | 4,7 | 3,7 | 9,2 | 4,2 | 37 | 45 | 11 | 56 | 19 |


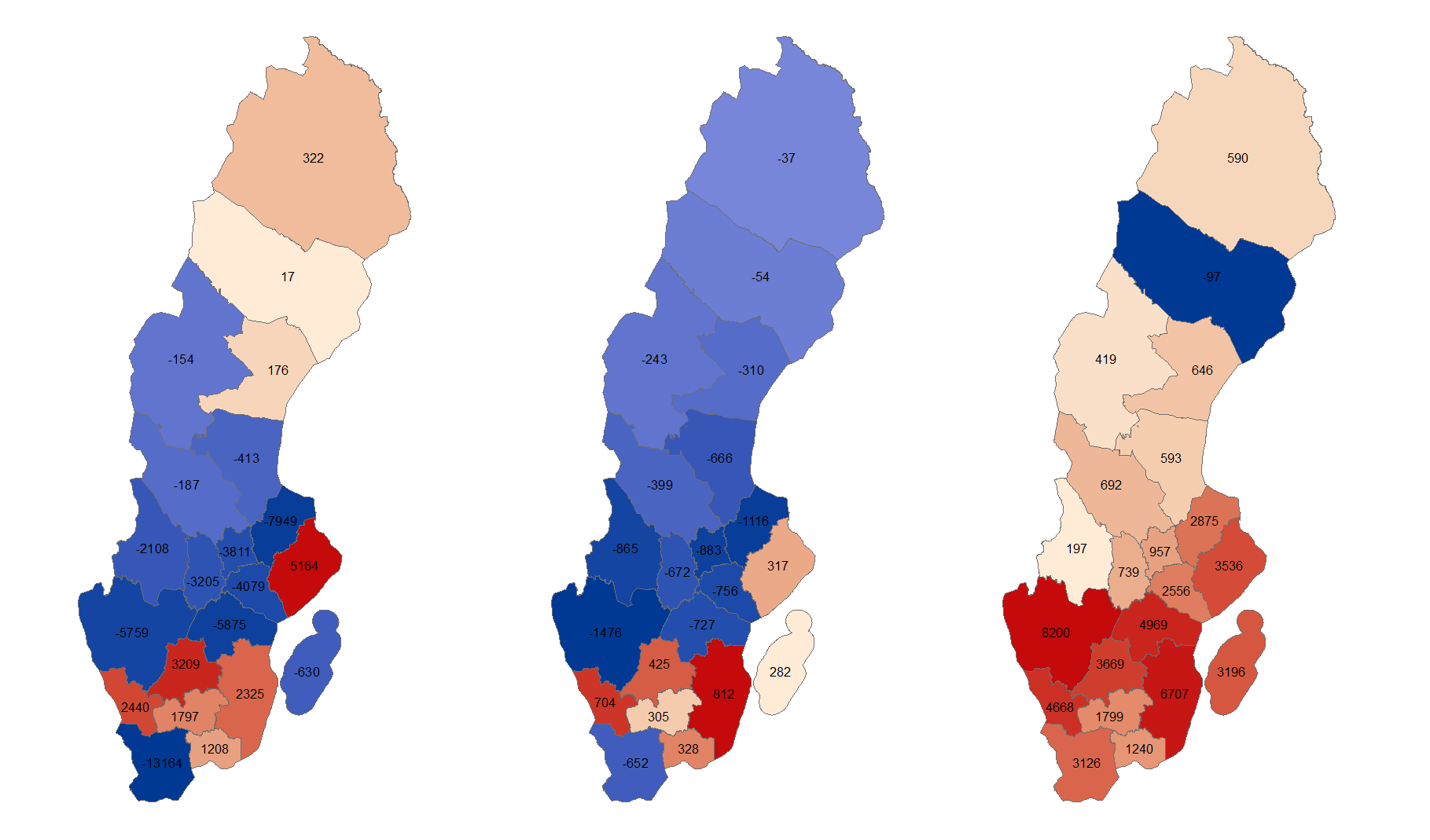


**Supplementary Figure S1:** Nutrient balance (Excreta supply minus crop nutrient need) of Swedish counties in 2007. Left N, middle P, and right K. Blue indicates a nutrient deficit and red indicates a nutrient surplus. Numbers in each county represent the surplus and deficit of the nutrient in tons.

**Supplementary Table S5**. Transport distances and P in excreta transported from surplus to deficit municipalities based on the P optimization model designed to minimize total national transport costs and eliminate surpluses of P in 2007 in Sweden Distances are the eucledian distance from the center points of municipalities times 1.33 to account for the road network length.

| Sent from municipality | Received in municipality | Distance (km) | Exchanged P (tons) |
| --- | --- | --- | --- |
| Ale | Vänersborg | 73 | 2 |
| Alvesta | Tidaholm | 197 | 54 |
| Alvesta | Gnosjö | 93 | 14 |
| Aneby | Hjo | 73 | 6 |
| Aneby | Ödeshög | 53 | 23 |
| Arjeplog | Sorsele | 100 | 1 |
| Bollebygd | Lidköping | 126 | 1 |
| Borgholm | Norrköping | 231 | 125 |
| Borgholm | Söderköping | 198 | 57 |
| Borgholm | Åtvidaberg | 188 | 3 |
| Borgholm | Valdemarsvik | 165 | 7 |
| Borås | Lidköping | 123 | 61 |
| Botkyrka | Ekerö | 31 | 22 |
| Boxholm | Vadstena | 49 | 11 |
| Bromölla | Eslöv | 103 | 35 |
| Båstad | Höganäs | 26 | 5 |
| Danderyd | Sigtuna | 38 | 17 |
| Eksjö | Vadstena | 124 | 54 |
| Emmaboda | Töreboda | 321 | 21 |
| Falkenberg | Vara | 193 | 100 |
| Falkenberg | Essunga | 181 | 11 |
| Falkenberg | Vårgårda | 152 | 22 |
| Falköping | Skara | 35 | 68 |
| Gislaved | Vara | 151 | 66 |
| Gotland | Nyköping | 213 | 195 |
| Gotland | Flen | 265 | 3 |
| Gotland | Gnesta | 248 | 22 |
| Gotland | Södertälje | 238 | 22 |
| Gotland | Nynäshamn | 183 | 18 |
| Gotland | Trosa | 190 | 14 |
| Gotland | Nykvarn | 255 | 6 |
| Gotland | Haninge | 197 | 2 |
| Gällivare | Boden | 196 | 9 |
| Gävle | Sandviken | 56 | 17 |
| Göteborg | Lidköping | 166 | 110 |
| Göteborg | Mellerud | 163 | 53 |
| Göteborg | Färgelanda | 145 | 87 |
| Göteborg | Tanum | 156 | 13 |
| Halmstad | Helsingborg | 95 | 60 |
| Halmstad | Höganäs | 68 | 49 |
| Hammarö | Karlstad | 36 | 6 |
| Herrljunga | Vara | 35 | 20 |
| Huddinge | Ekerö | 35 | 23 |
| Huddinge | Upplands-Bro | 54 | 22 |
| Hultsfred | Motala | 189 | 2 |
| Hylte | Vara | 189 | 9 |
| Härjedalen | Berg | 83 | 1 |
| Härryda | Lidköping | 143 | 15 |
| Hässleholm | Svalöv | 61 | 174 |
| Hässleholm | Bjuv | 66 | 19 |
| Högsby | Motala | 230 | 27 |
| Högsby | Vadstena | 212 | 5 |
| Hörby | Eslöv | 30 | 81 |
| Hörby | Lund | 39 | 26 |
| Höör | Kävlinge | 52 | 24 |
| Jokkmokk | Boden | 200 | 2 |
| Järfälla | Enköping | 61 | 34 |
| Jönköping | Skövde | 94 | 81 |
| Kalmar | Motala | 300 | 92 |
| Karlshamn | Kävlinge | 172 | 21 |
| Karlshamn | Lomma | 170 | 7 |
| Karlskrona | Trelleborg | 239 | 114 |
| Kinda | Linköping | 56 | 58 |
| Kiruna | Överkalix | 274 | 7 |
| Kiruna | Pajala | 176 | 4 |
| Klippan | Landskrona | 49 | 30 |
| Kristianstad | Eslöv | 73 | 189 |
| Kristianstad | Staffanstorp | 96 | 123 |
| Kristianstad | Lomma | 105 | 1 |
| Kungsbacka | Vänersborg | 159 | 41 |
| Kungälv | Munkedal | 104 | 9 |
| Laholm | Helsingborg | 74 | 128 |
| Laholm | Ängelholm | 40 | 23 |
| Lerum | Lidköping | 122 | 12 |
| Lerum | Vänersborg | 94 | 2 |
| Lessebo | Töreboda | 295 | 13 |
| Lidingö | Knivsta | 62 | 23 |
| Ljungby | Vara | 220 | 5 |
| Ljungby | Helsingborg | 141 | 27 |
| Ljungby | Bjuv | 133 | 13 |
| Ludvika | Ljusnarsberg | 39 | 2 |
| Luleå | Kalix | 55 | 1 |
| Lycksele | Vindeln | 80 | 2 |
| Lysekil | Tanum | 57 | 4 |
| Malmö | Lidköping | 443 | 54 |
| Malmö | Götene | 449 | 50 |
| Malung-Sälen | Torsby | 63 | 3 |
| Mark | Grästorp | 128 | 50 |
| Markaryd | Landskrona | 117 | 13 |
| Mullsjö | Götene | 98 | 3 |
| Mölndal | Lidköping | 159 | 29 |
| Mönsterås | Motala | 254 | 59 |
| Mönsterås | Finspång | 264 | 2 |
| Mörbylånga | Linköping | 309 | 47 |
| Mörbylånga | Motala | 356 | 28 |
| Mörbylånga | Ystad | 262 | 48 |
| Mörbylånga | Mjölby | 313 | 46 |
| Mörbylånga | Åtvidaberg | 280 | 30 |
| Nacka | Norrtälje | 92 | 45 |
| Nybro | Töreboda | 304 | 58 |
| Nybro | Karlsborg | 281 | 2 |
| Nässjö | Skövde | 134 | 7 |
| Nässjö | Hjo | 102 | 47 |
| Olofström | Landskrona | 158 | 5 |
| Olofström | Kävlinge | 155 | 16 |
| Orsa | Ovanåker | 75 | 5 |
| Orust | Munkedal | 68 | 2 |
| Osby | Bjuv | 111 | 30 |
| Oskarshamn | Katrineholm | 241 | 22 |
| Oxelösund | Flen | 92 | 6 |
| Partille | Lidköping | 141 | 18 |
| Perstorp | Landskrona | 64 | 24 |
| Robertsfors | Skellefteå | 92 | 38 |
| Robertsfors | Vindeln | 99 | 7 |
| Robertsfors | Vännäs | 85 | 7 |
| Ronneby | Lomma | 198 | 19 |
| Ronneby | Burlöv | 199 | 2 |
| Rättvik | Ovanåker | 50 | 1 |
| Salem | Strängnäs | 50 | 1 |
| Simrishamn | Trelleborg | 102 | 69 |
| Simrishamn | Ystad | 48 | 33 |
| Simrishamn | Skurup | 73 | 46 |
| Sjöbo | Svedala | 43 | 120 |
| Sjöbo | Lund | 31 | 32 |
| Sollentuna | Sigtuna | 30 | 3 |
| Sollentuna | Knivsta | 45 | 30 |
| Solna | Enköping | 78 | 9 |
| Solna | Sigtuna | 42 | 26 |
| Sotenäs | Tanum | 41 | 1 |
| Stenungsund | Vänersborg | 69 | 9 |
| Stockholm | Enköping | 82 | 128 |
| Stockholm | Strängnäs | 70 | 262 |
| Stockholm | Upplands-Bro | 40 | 12 |
| Stockholm | Håbo | 58 | 35 |
| Sundbyberg | Enköping | 75 | 19 |
| Svenljunga | Vara | 127 | 15 |
| Sävsjö | Skövde | 171 | 23 |
| Sävsjö | Tidaholm | 131 | 7 |
| Sävsjö | Habo | 103 | 2 |
| Sölvesborg | Trelleborg | 160 | 72 |
| Sölvesborg | Vellinge | 182 | 73 |
| Tibro | Töreboda | 33 | 3 |
| Tingsryd | Skövde | 293 | 4 |
| Tingsryd | Töreboda | 322 | 62 |
| Tjörn | Tanum | 103 | 6 |
| Tomelilla | Skurup | 52 | 61 |
| Torsås | Ystad | 228 | 47 |
| Tranemo | Vara | 112 | 24 |
| Tranås | Vadstena | 59 | 16 |
| Tranås | Ödeshög | 31 | 20 |
| Tyresö | Norrtälje | 98 | 23 |
| Täby | Vallentuna | 20 | 30 |
| Uddevalla | Färgelanda | 47 | 1 |
| Uddevalla | Munkedal | 40 | 2 |
| Ulricehamn | Vara | 72 | 62 |
| Umeå | Vännäs | 55 | 6 |
| Umeå | Nordmaling | 70 | 6 |
| Upplands Väsby | Sigtuna | 19 | 12 |
| Uppvidinge | Töreboda | 261 | 14 |
| Vaggeryd | Skövde | 143 | 16 |
| Varberg | Vänersborg | 193 | 53 |
| Varberg | Grästorp | 177 | 84 |
| Varberg | Essunga | 158 | 66 |
| Varberg | Trollhättan | 157 | 24 |
| Varberg | Alingsås | 125 | 24 |
| Varberg | Lilla Edet | 145 | 10 |
| Vaxholm | Vallentuna | 29 | 4 |
| Vetlanda | Hjo | 151 | 46 |
| Vilhelmina | Strömsund | 117 | 2 |
| Vimmerby | Linköping | 103 | 49 |
| Värmdö | Norrtälje | 79 | 14 |
| Värmdö | Österåker | 51 | 2 |
| Värnamo | Skövde | 190 | 11 |
| Värnamo | Skara | 189 | 15 |
| Västervik | Norrköping | 113 | 36 |
| Växjö | Skövde | 235 | 67 |
| Ydre | Vadstena | 92 | 45 |
| Åstorp | Bjuv | 12 | 3 |
| Älmhult | Bjuv | 126 | 20 |
| Älvkarleby | Tierp | 31 | 3 |
| Öckerö | Tanum | 140 | 7 |
| Örkelljunga | Bjuv | 50 | 22 |
| Östra Göinge | Svalöv | 96 | 3 |
| Östra Göinge | Landskrona | 123 | 53 |

**Supplementary Table S6**. Literature comparison of N and P balances in Swedish agriculture

| **Publication**  **/database** | **Balance Year** | **N kg/ha** | **P kg/ha** | **Input** | **Output** | **Remarks** |
| --- | --- | --- | --- | --- | --- | --- |
| Ulen et al. (2007)^1^ | 2004 | - | 2 | manure, mineral fertilizers, human excreta, atmospheric deposition | removal of nutrients with the harvest of crops | our estimate shows the same per ha P balance |
| Bergström et al. (2015)^2^ | 2007 | - | 2 | consumption of fertilizers, the gross input of manure | removal of nutrients with the harvest of crops | our estimate shows the same per ha P balance |
| Eurostat (2018)^3^ | 2007 | 45 | 1 | consumption of fertilizers, the gross input of manure, and other inputs. | removal of nutrients with the harvest of crops, removal of nutrients through the harvest and grazing of fodder, crop residues removed from the field. | N 3 kg/ha above and P 1 kg/ha less compared to our estimate |
| Statistics Sweden (2011)^4^ | 2007 | 39 | 3 | soil conditioners, seed, manure,  synthetic fertilizers, atmospheric deposition, legume fixation | removal of nutrients with the harvest of crops, removal of nutrients through the harvest and grazing of fodder, crop residues removed from the field. | N 3 kg/ha less and P 1 kg/ha above compared to our estimate |
| Linderholm et al., (2012)^5^ | 2008-2010 | - | 4.1 | synthetic fertilizers,  other fertilizers, imported fodder, feed minerals, live animals, atmospheric deposition, imported food, fish seafood & game animals | export of food, losses from arable land, household food waste, food industry waste, export of by-products of ethanol production, emissions from wastewater treatment plants, emissions from private sewage, sludge to non-agricultural use | P 2.1 kg/ha above compared to our estimate  The P balance was based on LCA analysis |
| van Dijk et al. (2016)^6^ | 2005 | - | 0.5 | the input of p: in crop production, animal production, food processing, non-food production | the output of p: in crop production, animal production, food processing, non-food production.  losses of p: in crop production, animal production, food processing, non-food production, human food consumptions | P 1.5 kg/ha less compared to our estimate.  the balance was based on the p flows in food consumption, production, waste-chain, and non-food |
| **This study** | **2007** | **42** | **2** | **livestock excreta**  **mineral fertilizers**  **human excreta** | **detailed spatial crop fertilizer recommendation**  **detailed spatial soil nutrient concentration** |  |

**Section 2: Results when we considered only the crop available nutrients in excreta**

**2.1 National nutrient supply and crop needs**

- Excreta can meet 52% of N and 67% of P crop needs but represents a K surplus (36 % over crop needs (Fig. S2).
- The national surplus that results from adding synthetic fertilizers and excreta is smaller than when total nutrients in excreta is considered, i.e., 63819 tons N surplus (33 % above crop needs), a 1791 tons of P surplus (6 % above crop needs), and a 64425 ton surplus of K (84% above crop needs) across Sweden (Fig. S2).
- At national scale these surpluses translate to an excess of 25 kg per ha of N, 0.70 kg per ha of P, and 25 kg per ha of K.


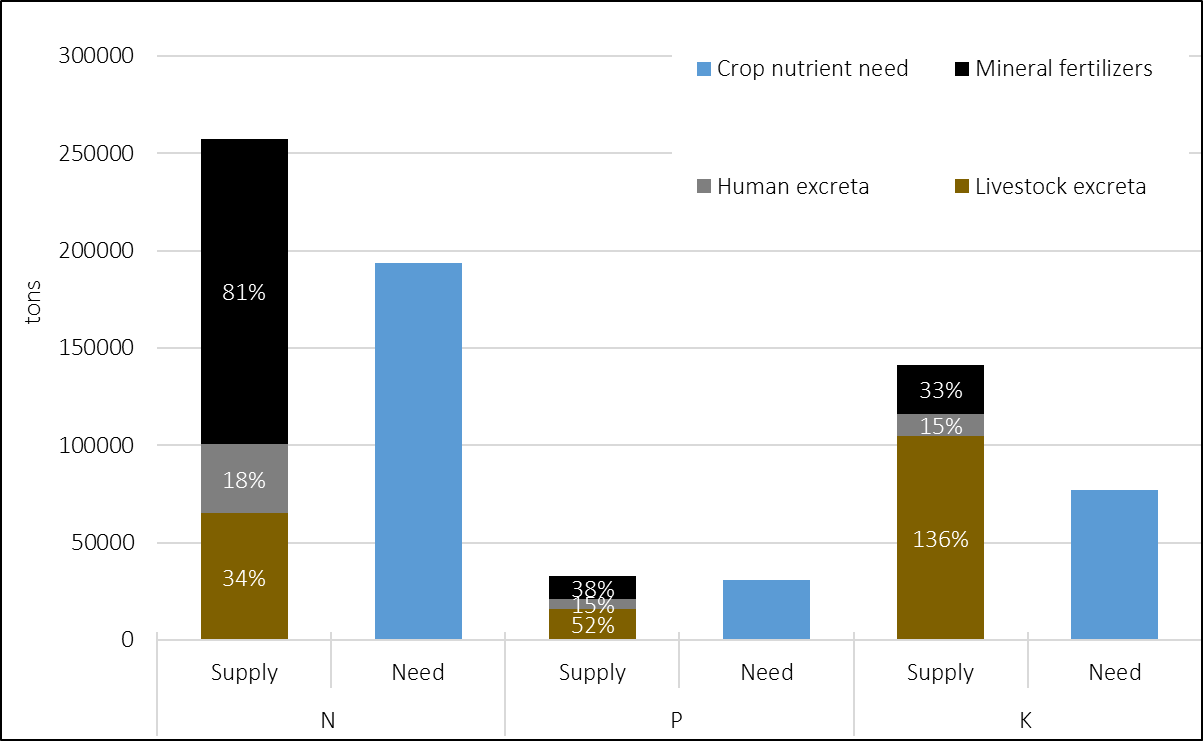


**Supplementary Figure S2**. National 2007 Swedish crop available nutrient supply and crop nutrient need. Nutrient supply sources (livestock and human excreta and synthetic fertilizers) are presented as both total amount of nutrients (high of bars) and as a percentage of total crop nutrient needs (white numbers in bars).

**2.2 Municipal nutrient balances**

- Only 17% of municipalities would have more N in excreta than crop nutrient need; while 34% would have P surpluses, and 76% would have K surpluses (Table S8).
- Recycling excreta *within* municipalities could meet 47 % of N, 56 % P, and 91 % K crop needs in Sweden.
- Transporting excreta from surplus municipalities towards deficit municipalities could meet an additional 5% of N, 12% of P, and 60% of K national crop nutrient needs (Table S8).

**Supplementary Table S7**. Minimum, maximum, and average crop nutrient needs and crop available excreta supply in Swedish municipalities in 2007.

|  | N (kg per ha) | | | P (kg per ha) | | | K (kg per ha) | | |
| --- | --- | --- | --- | --- | --- | --- | --- | --- | --- |
|  | Min | Max | Avg | Min | Max | Avg | Min | Max | Avg |
| Crop need | 0 | 103 | 75 | 0 | 29 | 12 | 0 | 103 | 30 |
| Livestock excreta supply | 0 | 78 | 25 | 0 | 19 | 6 | 0 | 143 | 41 |
| Human excreta supply | 2 | 245041 | 14 | 0 | 32925 | 2 | 0 | 80414 | 5 |
| Total nutrients as excreta | 11 | 245041 | 39 | 2 | 32925 | 8 | 6 | 80414 | 45 |


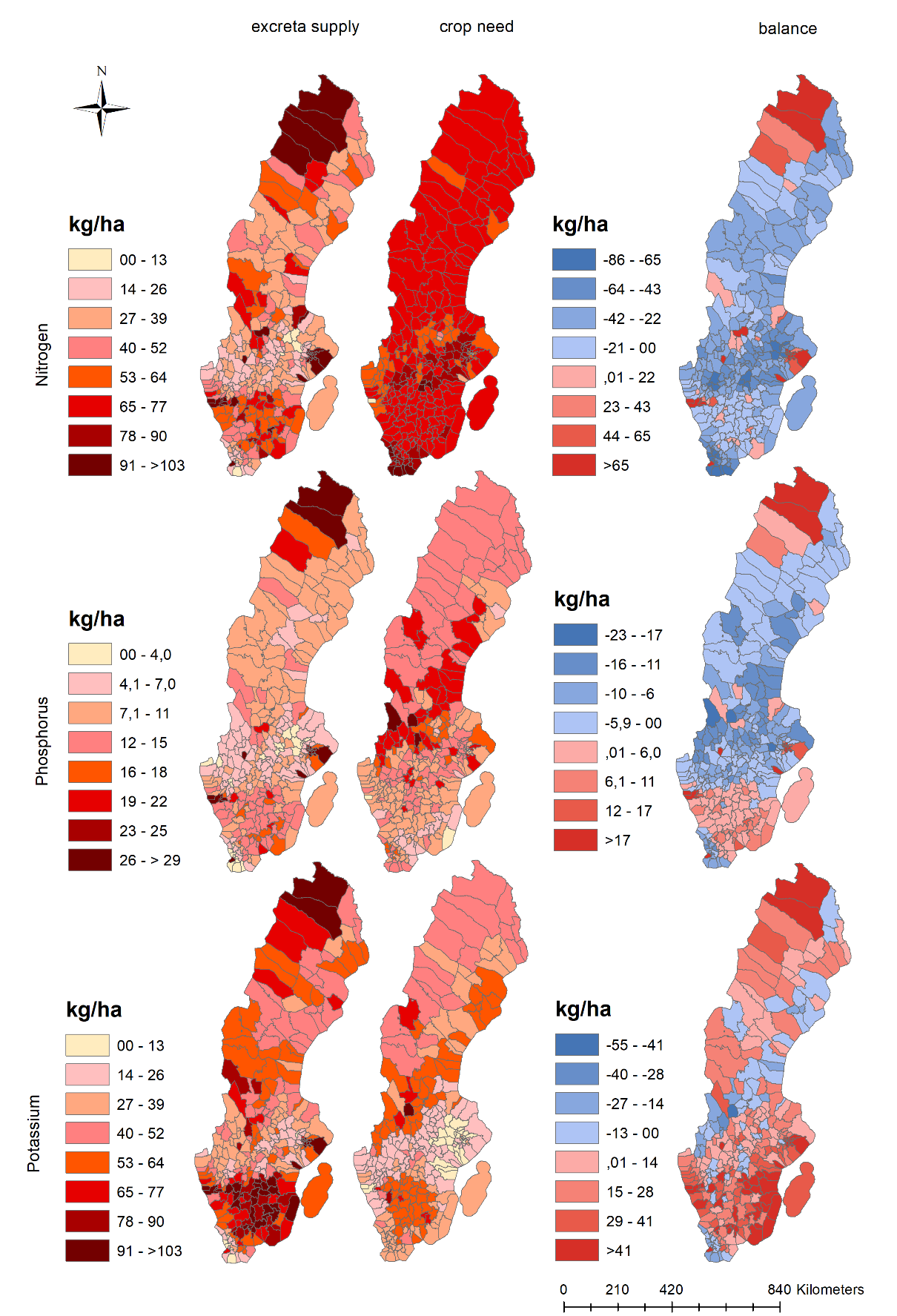


**Supplementary Figure S3**. Spatial distribution of crop available nutrients in excreta, crop nutrient needs, and nutrient balances of Swedish municipalities. The top panel represents N, the middle P, and the bottom K distributions. The right-side balance maps are created by subtracting crop nutrient need (middle) from excreta nutrient supply (left) in each case. Note that although the color scales are the same for all three nutrients, the values associated with each color are not (e.g., N values are much higher than for P).


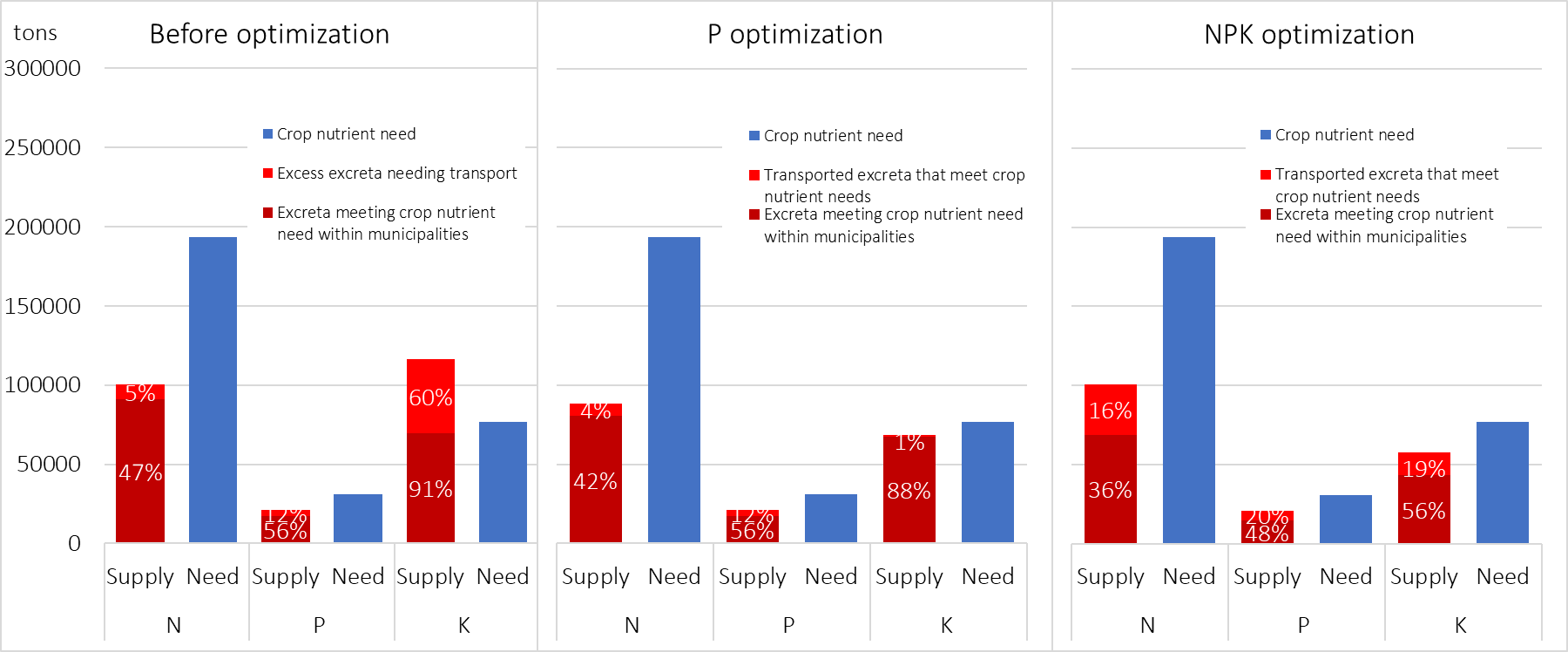


**Supplementary Figure S4**. Crop available nutrient in excreta that can be recycled within municipalities and transported between municipalities to meet crop needs. A) The amount of crop nutrient needs that could theoretically be met by recycling before any optimization is run. B) The amount of crop nutrient needs that could be met based on the redistribution made by the P optimization model. The amount of crop nutrient needs that could be met based on the redistribution made by the NPK optimization model. Values are presented as the total amount of plant available nutrients (y-axis) and as a percentage of total crop nutrient needs (white numbers in bars). Panels B and C only show the amount of excreta that meets crop nutrient needs, and not the surplus amounts within municipalities or resulting from excreta transport (all numbers are available in Table S12).

**Supplementary Table S8**. Breakdown of municipalities with surplus and deficits of nutrients in relation to their share of arable land, crop nutrient needs, and crop available nutrients in excreta at the national level. The net balance is the difference between crop needs and nutrients available in excreta, which is also represented as surplus percentage (%) of crop need and deficit percentage (%) of crop need.

|  |  | N | | P | | K | |
| --- | --- | --- | --- | --- | --- | --- | --- |
| Municipality balance |  | Surplus | Deficit | Surplus | Deficit | Surplus | Deficit |
| No. Of Municipalities |  | 50 | 240 | 99 | 191 | 221 | 69 |
| Arable Land | (1000) Ha | 69 | 2510 | 722 | 1856 | 1896 | 682 |
|  | % Of Total | 3 | 97 | 28 | 72 | 74 | 26 |
| Crop Need | Tons | 4807 | 188862 | 6114 | 24873 | 51358 | 25564 |
|  | Kg/Ha | 70 | 75 | 8 | 13 | 27 | 37 |
|  | % Of Total | 2 | 98 | 20 | 80 | 67 | 33 |
| Livestock excreta supply | Tons | 2201 | 62828 | 7530 | 8674 | 88157 | 16477 |
|  | Kg/Ha | 32 | 25 | 10 | 5 | 46 | 24 |
|  | % Of Total | 3 | 97 | 46 | 54 | 84 | 16 |
| Human excreta Supply | Tons | 12163 | 23375 | 2300 | 2475 | 9639 | 2024 |
|  | Kg/Ha | 177 | 9 | 3 | 1 | 5 | 3 |
|  | % Of Total | 34 | 66 | 48 | 52 | 83 | 17 |
| Total nutrients as excreta | Tons | 14364 | 86203 | 9830 | 11149 | 97796 | 18501 |
|  | Kg/Ha | 209 | 34 | 14 | 6 | 52 | 27 |
|  | % Of Total | 14 | 86 | 47 | 53 | 84 | 16 |
| Net balance | Tons | 9558 | -102659 | 3716 | -13724 | 46438 | -7063 |
|  | Kg/Ha | 139 | -41 | 5 | -7 | 24 | -10 |
|  | % Of national crop need | 5 | -53 | 12 | -44 | 60 | -9 |

**2.3 Transportation to redistribute surpluses**

- Transporting excess excreta from surplus municipalities would require 15,984 km of truck travel (Table S12).
- The average export distance between a surplus and a deficit municipality is 163 km (Table S9).
- The cost of transporting surplus excreta-P to meet crop needs is eight times the market value of P fertilizers it would replace, but only 3.8 times higher than the total N:P:K fertilizer value being transported. This is slightly higher than the value obtained in the main analysis which uses the total amount of nutrient in excreta (Table S12).
- Swedish farmers could purchase 60% of N, 85% of P, and 25% of K less synthetic fertilizers than they did in 2007 by reusing excreta optimally even if we only consider crop available nutrients.

**Supplementary Table S9**. Summary values of transport amounts (tons of crop available P in excreta) and distances (km) optimized to minimize costs (distance x fresh weight) to meet P crop needs. Export/import connections represent the number of municipalities a surplus or deficit municipality would export to or import from.

|  | Export (tons) | Import (tons) | Export distance (km) | Import distance (km) | Export connections | Import connections |
| --- | --- | --- | --- | --- | --- | --- |
| Min | 1 | 1 | 19 | 16 | 1 | 1 |
| Max | 413 | 335 | 1080 | 1198 | 8 | 7 |
| Average | 38 | 49 | 163 | 210 | 2 | 2 |


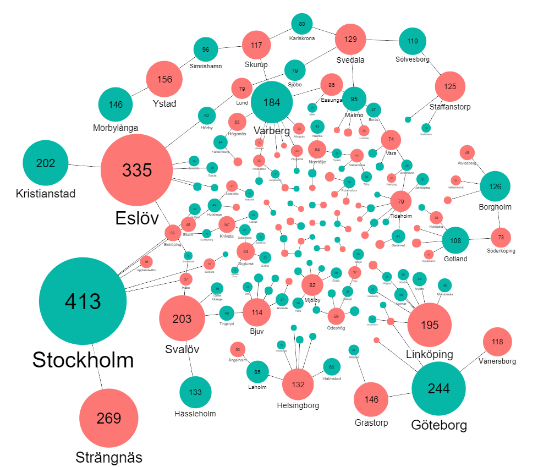

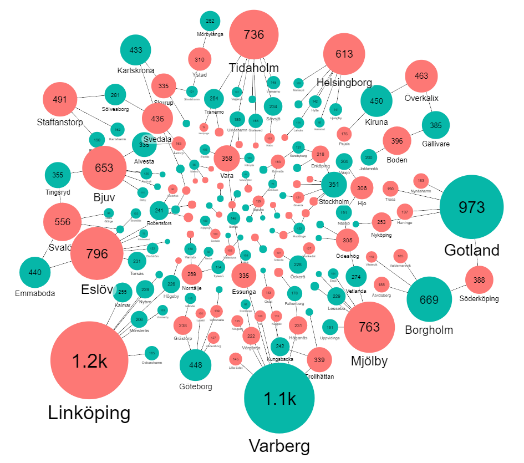


**Supplementary Figure S5**: Transport network of crop available excreta-P from surplus (sea green) to deficit (brick red) municipalities based on P optimization model outputs to minimize total national transport distance and eliminate surpluses of P. The left panel shows the amount (tons) of crop available excreta-P exported or imported from or to a municipality where bubble size is proportional to the amount (also expressed as the number in the bubble) . The right panel shows the distance (km) excreta-P traveled from/to a municipality.

**Section 3: P optimization model vs NPK optimization model**

**3.1 Summary of NPK optimization model results using total nutrient amount in excreta**

- A total of 9.5 million tons of excreta would be moved among the municipalities (1.8 times the amount in the P optimization model) covering a total distance of 53, 463 km of truck travel which is 2.2 times the distance required in the P optimization model (Table S12). The average export distance is 294 km (Table S10).
- There is no overapplication of N and P, but 17, 263 tons of transported K are still in excress of crop needs (Table S12), which is much lower than the P optimization model where 96% of K and 55% of N transported was actually overapplied.
- This transportation of larger amounts of useful nutrients results in a better ratio (1.3) of transport cost to market value of N:P:K fertilizers it would replace, than the ratio (3.68) from the P optimization model (Table S12).
- After the NPK optimization, 98 municipalities were balanced in terms of N, 167 in terms of P and only 11 in terms of K (Table S13).
- Sweden could purchase as little as 31% of N, 48% of P, and 13% of K of its 2007 synthetic fertilizer use, which is 6% and 4% less for these reductions from the P optimization model.

**Supplementary Table S10.** Summary values of transport amounts (tons of NPK) and distances (km) optimized to minimize costs (distance x fresh weight) to meet NPK crop needs, when used NPK optimization model. Export/import connections represent the number of municipalities a surplus or deficit municipality would export to or import from.

|  | Export (tons) | | | Import (tons) | | |  |  |  |  |
| --- | --- | --- | --- | --- | --- | --- | --- | --- | --- | --- |
|  | N | P | K | N | P | K | Export distance (km) | Import distance (km) | Export connections | Import connections |
| Min | 1 | 0 | 1 | 5 | 1 | 3 | 12 | 19 | 1 | 1 |
| Max | 3298 | 439 | 1711 | 1985 | 320 | 2060 | 3145 | 1652 | 12 | 8 |
| Average | 347 | 59 | 280 | 309 | 53 | 250 | 294 | 262 | 2 | 2 |

**3.2 Summary of NPK optimization model results using crop available nutrients in excreta**

- A total of 5.9 million tons of excreta would be moved among the municipalities (1.7 times the amount transported in the P optimization model for crop available nutrients in excreta) covering a total distance of 22, 412 km of truck travel (1.4 times the amount in the P optimization model for crop available nutrients in excreta; Table S12). The average export distance is 165 km, which is almost equal to the average export distance in the P optimization model run for crop available nutrients in excreta (Table S11).
- There is no overapplication of N and, but about 8, 000 tons of transported K are in excess of crop needs (Table S12), which is lower the P optimization model run for crop available nutrients in excreta where 95% of K amd 60% of N transporetd was actually overapplied.
- The transportaion of a larger amount of ‘ useful’ nutrients results in a better ratio (1.2) of transport to market value of NPK fertilizers transported than the one obtained from the P optimization model using crop available nutrients in excreta (3.8 ratio, Table S12).
- Sweden would only need to purchase 59% of N, 85% of P, and 22% of K of its 2007 use of synthetic fertilizers in crop production (as tons).

**Supplementary Table S11.** Summary values of transport amounts (tons of crop available NPK in excreta) and distances (km) optimized to minimize costs (distance x fresh weight) to meet NPK crop needs, when used NPK optimization model. Export/import connections represent the number of municipalities a surplus or deficit municipality would export to or import from.

|  | Export (tons) | | | Import (tons) | | |  |  |  |  |
| --- | --- | --- | --- | --- | --- | --- | --- | --- | --- | --- |
|  | N | P | K | N | P | K | Export distance (km) | Import distance (km) | Export connections | Import connections |
| Min | 1 | 0 | 1 | 3 | 1 | 3 | 10 | 20 | 1 | 1 |
| Max | 3080 | 414 | 1018 | 1963 | 335 | 1864 | 1027 | 897 | 8 | 9 |
| Average | 232 | 44 | 223 | 249 | 48 | 239 | 165 | 176 | 2 | 2 |

**3.3 Comparison of all modeled scenarios**

**Supplementary Table S12:** Summary of optimized excreta transports for the P optimization and NPK optimization models to meet crop nutrient needs in 2007 at the municipal scale in Sweden.

| model | Scenario | Total distance (km) | total excreta transported (000 tons) | Transported nutrient meeting crop need (000 tons) | | | Transported nutrient not meeting crop need (waste) (000 tons) | | | Value of correctly transported nutrient (M USD) | Transport cost (M USD) | Transport cost times of the value |
| --- | --- | --- | --- | --- | --- | --- | --- | --- | --- | --- | --- | --- |
|  |  |  |  | N | P | K | N | P | K |  |  |  |
| P optimization | crop available nutrients in excreta | 15984 | 3592 | 7,99 | 3,71 | 0,68 | 11,76 | 0,00 | 17,26 | 29 | 111 | 3,81 |
|  | total nutrients in excreta | 24079 | 5345 | 15,78 | 5,92 | 1,23 | 19,26 | 0,00 | 26,86 | 52 | 192 | 3,68 |
| NPK optimization | crop available nutrients in excreta | 22412 | 5931 | 31,62 | 6,05 | 22,17 | 0,00 | 0,00 | 8,13 | 103 | 124 | 1,20 |
|  | total nutrient in excreta | 53463 | 9535 | 63,10 | 10,71 | 33,74 | 0,00 | 0,00 | 17,26 | 190 | 240 | 1,27 |

**Supplementary Table S13:** Municipalities with a balance, surplus or deficit of each nutrient after optimized excreta transports for the P optimization and NPK optimization models to meet crop nutrient needs at municipal scale in 2007 in Sweden.

| model | Scenario | Balanced | | | Surplus | | | Deficit | | |
| --- | --- | --- | --- | --- | --- | --- | --- | --- | --- | --- |
|  |  | N | P | K | N | P | K | N | P | K |
| P optimization | crop available nutrients in excreta | 8 | 160 | 7 | 35 | 0 | 215 | 247 | 130 | 68 |
|  | total nutrient in excreta | 9 | 193 | 8 | 69 | 0 | 230 | 212 | 97 | 52 |
| NPK optimization | crop available nutrients in excreta | 35 | 126 | 11 | 0 | 7 | 218 | 255 | 157 | 61 |
|  | total nutrient in excreta | 98 | 167 | 11 | 6 | 4 | 236 | 186 | 119 | 43 |

**Section 4: Methods**

**4.1 Crop available nutrients in excreta**

Using the total amount of nutrients in excreta is a common and well documented practice to calculate nutrient budgets (OECD & Eurostat, 2007); however depending on how excreta is collected, stored, and processed, it is likely that the total amount of nutrients in excreta are not usable by crops in the first year after excreta has been applied to fields. Nutrients can change forms over time in soils and plant can use only some of these nutrients’ forms ^7^. Swedish law requires farmers to incorporate excreta into the soil within less than 12 hours after application ^8^. Complying with the law should mean that over 55% N in excreta (depending on the excreta type) is crop available in the first year of its application, while 80% of P and 90% of K remain available (Wilson 2018; Table S14).

**Supplementary Table S14:** Annual nutrient excretions, gaseous loss during storage, likely crop availability, fresh weight of excreta/sludge by individual livestock types and human in Sweden

|  | Kg per animal per year | | | % of the excreted nutrient content is available after storage (jakobsson and steineck 2012) | | | Nutrient content left at storage (kg). Values used to calculate total nutrient in excreta | | | % of the nutrient content applied to soil is crop available (jonsson et al. 2004; wilson 2018) | | | Crop available nutrients (kg). Values used to calculate crop available nutrients in excreta | | | Excreta (kg) |
| --- | --- | --- | --- | --- | --- | --- | --- | --- | --- | --- | --- | --- | --- | --- | --- | --- |
| TYPE | N | P | K | N | P | K | N | P | K | N | P | K | N | P | K |  |
| DAIRY COWS | 117,00 | 16,00 | 104,00 | 90 | 100 | 100 | 105,3 | 16 | 104 | 55 | 80 | 90 | 57,92 | 12,80 | 93,60 | 26088 |
| COWS FOR CALF PRODUCTION | 63,00 | 12,00 | 75,00 | 90 | 100 | 100 | 56,7 | 12 | 75 | 60 | 80 | 90 | 34,02 | 9,60 | 67,50 | 12194 |
| HEIFERS, BULLS AND STEERS | 47,00 | 8,00 | 54,00 | 90 | 100 | 100 | 42,3 | 8 | 54 | 60 | 80 | 90 | 25,38 | 6,40 | 48,60 | 10295 |
| CALVES, UNDER 1 YEAR | 21,00 | 3,00 | 28,00 | 90 | 100 | 100 | 18,9 | 3 | 28 | 60 | 80 | 90 | 11,34 | 2,40 | 25,20 | 5998 |
| SHEEPS | 14,00 | 2,00 | 19,00 | 80 | 100 | 100 | 11,2 | 2 | 19 | 60 | 80 | 90 | 6,72 | 1,60 | 17,10 | 797 |
| BREEDING BOARS | 27,30 | 5,40 | 9,70 | 90 | 100 | 100 | 24,57 | 5,4 | 9,7 | 75 | 80 | 90 | 18,43 | 4,32 | 8,73 | 3197 |
| BREEDING SOWS | 36,00 | 10,00 | 13,00 | 90 | 100 | 100 | 32,4 | 10 | 13 | 75 | 80 | 90 | 24,30 | 8,00 | 11,70 | 7796 |
| FATTENING PIGS, 20 KG AND OVER | 11,00 | 2,30 | 4,30 | 90 | 100 | 100 | 9,9 | 2,3 | 4,3 | 75 | 80 | 90 | 7,43 | 1,84 | 3,87 | 2599 |
| POULTRY | 0,22 | 0,06 | 0,06 | 90 | 100 | 100 | 19,8 | 0,06 | 0,06 | 70 | 80 | 90 | 0,1386 | 0,048 | 0,054 | 2498 |
| LAYING CHICKENS | 0,52 | 0,13 | 0,17 | 90 | 100 | 100 | 46,8 | 0,13 | 0,17 | 70 | 80 | 90 | 0,3276 | 0,104 | 0,153 | 9595 |
| BROILERS | 0,28 | 0,06 | 0,11 | 80 | 100 | 100 | 22,4 | 0,06 | 0,11 | 70 | 80 | 90 | 0,1568 | 0,048 | 0,099 | 694 |
| TURKIES | 0,69 | 0,24 | 0,31 | 80 | 100 | 100 | 55,2 | 0,24 | 0,31 | 70 | 80 | 90 | 0,3864 | 0,192 | 0,279 | 1686 |
| HORSES | 48,00 | 9,00 | 58,00 | 80 | 100 | 100 | 38,4 | 9 | 58 | 60 | 80 | 90 | 23,04 | 7,20 | 52,20 | 4940 |
| HUMAN EXCRETA* | 4,60 | 0,55 | 1,30 | 90 | 100 | 100 | 4,14 | 0,55 | 1,3 | 93 | 95 | 98 | 3,87 | 0,52 | 1,27 | 32 |

*****N, P, and K in human urine has the same form as that of chemical fertilizers and thus is almost 100% available to crops ^10^. It is only for N that the availability of fecal nutrients is considerably lower than that of chemical fertilizers or urine ^10^. For fecal nutrients, we assumed that 50% of N and 80% of P and 90% of K is crop available in the first year of its application.

**4.2 Fertilizer needs (nutrient recommendation in kg/ha)**

**Supplementary Table S15.** Fertilizer needs (nutrient recommendation in kg/ha) to produce crops in Sweden according to soil type. These values are used to calculate crop nutrient needs.

|  | Nitrogen (kg/ha) | | | Phosphorus (kg/ha) | | | | | | | Potassium (kg/ha) | | | | | |
| --- | --- | --- | --- | --- | --- | --- | --- | --- | --- | --- | --- | --- | --- | --- | --- | --- |
|  | Production region | | | Soil class P-AL | | | | | | | Soil class K-AL | | | | | |
| Crop | södra Götaland | norra Götaland & Svealand | Norrland | I | II | III | IVA | IVB | V | AVG. | I | II | III | IV | V | AVG. |
| winter wheat | 130 | 145 | 145 | 38 | 28 | 18 | 13 | 8 | 0 | 18 | 70 | 50 | 30 | 10 | 0 | 32 |
| spring wheat | 105 | 105 | 105 | 32 | 22 | 12 | 7 | 2 | 0 | 13 | 60 | 40 | 20 | 0 | 0 | 24 |
| rye | 90 | 90 | 90 | 38 | 28 | 18 | 13 | 8 | 0 | 18 | 70 | 50 | 30 | 10 | 0 | 32 |
| winter barley | 75 | 80 | 80 | 35 | 25 | 15 | 10 | 5 | 0 | 15 | 65 | 45 | 25 | 5 | 0 | 28 |
| barley | 70 | 80 | 80 | 32 | 22 | 12 | 7 | 2 | 0 | 13 | 60 | 40 | 20 | 0 | 0 | 24 |
| spring barley | 70 | 80 | 80 | 32 | 22 | 12 | 7 | 2 | 0 | 13 | 60 | 40 | 20 | 0 | 0 | 24 |
| oats | 60 | 70 | 70 | 32 | 22 | 12 | 7 | 2 | 0 | 13 | 60 | 40 | 20 | 0 | 0 | 24 |
| triticale | 95 | 100 | 100 | 35 | 25 | 15 | 10 | 5 | 0 | 15 | 65 | 45 | 25 | 5 | 0 | 28 |
| mixed grain | 87 | 94 | 94 | 29 | 19 | 9 | 4 | 0 | 0 | 10 | 55 | 35 | 15 | 0 | 0 | 21 |
| peas vetches and beans | 0 | 0 | 0 | 34 | 24 | 14 | 9 | 4 | 0 | 14 | 75 | 55 | 35 | 5 | 0 | 34 |
| green peas | 0 | 0 | 0 | 34 | 24 | 14 | 9 | 4 | 0 | 14 | 75 | 55 | 35 | 5 | 0 | 34 |
| white beans | 0 | 0 | 0 | 32 | 22 | 12 | 7 | 2 | 0 | 13 | 70 | 50 | 30 | 0 | 0 | 30 |
| greenfodder | 115 | 115 | 115 | 25 | 18 | 10 | 5 | 0 | 0 | 10 | 120 | 90 | 60 | 15 | 0 | 57 |
| Utilized ley for hay | 80 | 80 | 80 | 32 | 22 | 12 | 7 | 2 | 0 | 13 | 110 | 70 | 30 | 0 | 0 | 42 |
| Utilized pasture | 23 | 23 | 23 | 25 | 15 | 5 | 0 | 0 | 0 | 8 | 60 | 40 | 20 | 0 | 0 | 24 |
| Ley for seeds | 50 | 50 | 50 | 30 | 23 | 15 | 8 | 4 | 0 | 13 | 100 | 75 | 50 | 25 | 0 | 50 |
| Table potatoes | 80 | 80 | 80 | 98 | 78 | 58 | 38 | 28 | 18 | 53 | 258 | 208 | 158 | 108 | 0 | 146 |
| Potatoes for processing | 80 | 80 | 80 | 100 | 80 | 60 | 40 | 30 | 20 | 55 | 260 | 210 | 160 | 110 | 0 | 148 |
| Sugar beets | 120 | 120 | 120 | 54 | 44 | 29 | 24 | 14 | 0 | 28 | 104 | 79 | 54 | 34 | 0 | 54 |
| Winter rape | 115 | 115 | 115 | 40 | 30 | 20 | 15 | 10 | 0 | 19 | 70 | 50 | 30 | 10 | 0 | 32 |
| Spring rape | 90 | 90 | 90 | 35 | 25 | 15 | 10 | 5 | 0 | 15 | 60 | 40 | 20 | 0 | 0 | 24 |
| Winter turnip rape | 90 | 90 | 90 | 33 | 23 | 13 | 8 | 3 | 0 | 13 | 55 | 35 | 15 | 0 | 0 | 21 |
| Spring turnip rape | 80 | 80 | 80 | 32 | 22 | 12 | 7 | 2 | 0 | 13 | 53 | 33 | 13 | 0 | 0 | 20 |
| Oil flax | 62 | 62 | 62 | 33 | 23 | 13 | 8 | 3 | 0 | 13 | 55 | 35 | 15 | 0 | 0 | 21 |
| Horticulture plants | 35 | 35 | 35 | 7 | 5 | 4 | 2 | 0 | 0 | 3 | 35 | 26 | 18 | 9 | 0 | 18 |
| Other crops | 87 | 87 | 87 | 35 | 25 | 15 | 10 | 5 | 0 | 15 | 59 | 39 | 19 | 2 | 0 | 24 |
| Energy forest | 160 | 160 | 160 | 30 | 25 | 20 | 10 | 5 | 0 | 15 | 80 | 65 | 50 | 25 | 0 | 44 |
| Fallow land | 0 | 0 | 0 | 0 | 0 | 0 | 0 | 0 | 0 | 0 | 0 | 0 | 0 | 0 | 0 | 0 |
| Other untilled arable land | 0 | 0 | 0 | 0 | 0 | 0 | 0 | 0 | 0 | 0 | 0 | 0 | 0 | 0 | 0 | 0 |
| Unspecified arable land | 111 | 111 | 111 | 56 | 42 | 28 | 14 | 7 | 0 | 25 | 242 | 182 | 121 | 61 | 0 | 121 |

1. Ulen, B., Folster, J., Bechmann, M., Jarvie, H. P. & Tunney, H. Agriculture as a phosphorus source for eutrophication in the north-west European countries, Norway, Sweden, United Kingdom and Ireland: a review. (Special issue: Agriculture, Phosphorus, Eutrophication: a European Perspective.). *Soil Use Manag.* **23,** 5–15 (2007).

2. Bergström, L. *et al.* Turnover and Losses of Phosphorus in Swedish Agricultural Soils: Long-Term Changes, Leaching Trends, and Mitigation Measures. *J. Environ. Qual.* **44,** 512 (2015).

3. Eurostat. Gross nutrient balance. (2018). Available at: http://appsso.eurostat.ec.europa.eu/nui/show.do?dataset=aei_pr_gnb&lang=en. Eurostat. (Accessed: 14th April 2019)

4. Statistics Sweden. Nitrogen and phosphorus balances for agricultural land and agricultural sector in 2007. (2011). Available at: https://www.scb.se/statistik/MI/MI1004/2007A03/MI1004_2007A03_SM_MI40SM1101.pdf.

5. Linderholm, K., Mattsson, J. E. & Tillman, A. M. Phosphorus flows to and from swedish agriculture and food chain. *Ambio* **41,** 883–893 (2012).

6. van Dijk, K. C., Lesschen, J. P. & Oenema, O. Phosphorus flows and balances of the European Union Member States. *Sci. Total Environ.* **542,** 1078–1093 (2016).

7. Wilson, M. Manure characteristics. (2018). Available at: https://extension.umn.edu/manure-land-application/manure-characteristics#nitrogen-817860. (Accessed: 4th April 2019)

8. Nilsson, A. K. *Regulating Zero Eutrophication. Swedish Law on Controlling Emis-sions of Nutrients into the Baltic Sea*. (2013).

9. Jakobsson, C. & Steineck, S. Management of manure on the Farm: One of the Keys to the Future. in *Sustainable Agriculture Ecosystem Health and Sustainable Agriculture 1* (ed. Jakobsson, C.) 505 (The Baltic University Programme, Uppsala University, 2012, 2012).

10. Jonsson, H., Stinzing, A. R., Vinneras, B. & Salomon, E. Guidelines on the Use of Urine and Faeces in Crop Production. *EcoSanRes Publ. Ser.* **2,** 1–35 (2004).
